# The fungal root endophyte *Serendipita vermifera* displays inter-kingdom synergistic beneficial effects with the microbiota in *Arabidopsis thaliana* and barley

**DOI:** 10.1038/s41396-021-01138-y

**Published:** 2021-10-22

**Authors:** Lisa K. Mahdi, Shingo Miyauchi, Charles Uhlmann, Ruben Garrido-Oter, Gregor Langen, Stephan Wawra, Yulong Niu, Rui Guan, Senga Robertson-Albertyn, Davide Bulgarelli, Jane E. Parker, Alga Zuccaro

**Affiliations:** 1 University of Cologne, Institute for Plant Sciences, Cologne, Germany; 2 Max Planck Institute for Plant Breeding Research, Department of Plant Microbe Interactions, Cologne, Germany; 3 Cluster of Excellence on Plant Sciences (CEPLAS), Cologne, Germany; 4 University of Dundee, Plant Sciences, School of Life Sciences, Dundee, UK

**Keywords:** Plant sciences, Symbiosis

## Abstract

Plant root-associated bacteria can confer protection against pathogen infection. By contrast, the beneficial effects of root endophytic fungi and their synergistic interactions with bacteria remain poorly defined. We demonstrate that the combined action of a fungal root endophyte from a widespread taxon with core bacterial microbiota members provides synergistic protection against an aggressive soil-borne pathogen in *Arabidopsis thaliana* and barley. We additionally reveal early inter-kingdom growth promotion benefits which are host and microbiota composition dependent. Using RNA-sequencing, we show that these beneficial activities are not associated with extensive host transcriptional reprogramming but rather with the modulation of expression of microbial effectors and carbohydrate-active enzymes.

## Introduction

Plant pathogenic fungi limit crop productivity globally. These threats are expected to increase with global warming [1]. Decades of advances in agrochemicals and plant breeding have expanded farmers’ toolkits with fungicides and resistant varieties to limit the detrimental effects of these organisms on crop yield. Yet, current tools are becoming environmentally unsustainable or ineffective against rapidly evolving pathogens [1]. A key example of this scenario is represented by the soil-borne plant pathogen *Bipolaris sorokiniana* (syn. *Cochliobolus sativus*, hereafter *Bs*), the causal agent of spot blotch and common root rot diseases that threaten cereal production in warm regions [1–3]. Root rot normally originates from inoculum carried on the seed or from soil-borne conidia, but the fungus can infect plants at any developmental stage. However, as the importance of root-inhabiting pathogenic fungi has often been underestimated, very little is known about the molecular mechanism behind the detrimental interaction of *Bs* with roots [4].

Microbial communities living at the root−soil interface, collectively referred to as the plant root microbiota, have gained centre-stage in pathogen protection [5]. Past studies across a variety of plant species employed environmental sampling or controlled conditions in the field and laboratory to characterize the root microbiota [6–10], with an overall greater focus on bacteria than on filamentous fungi [11]. Microbial diversity and abundance gradually decrease between the soil and vicinity of the root (rhizosphere), and further between the rhizosphere and root internal compartments (endosphere). Moreover, a number of bacterial taxa (e.g., Proteobacteria, Actinobacteria, Bacteroidetes, and Firmicutes) consistently occur in the root endosphere of different examined plant species [10]. This latter feature underpins the “bacterial core microbiota” concept, in which strains from specific taxa are commonly selected as endophytes across plant species, soil types, and environmental conditions [12]. By contrast, studies of geographically distinct populations of *Arabis alpina* and *Arabidopsis thaliana* (hereafter Arabidopsis) showed that few fungal taxa are prevalent in the root endosphere, and that endophytic fungal communities are strongly influenced by location and climate [9, 13].

The functions and benefits of root microbiota members in the context of abiotic or biotic stresses have been extensively investigated under laboratory conditions using single microbial strains and, more recently, synthetic bacterial communities (SynComs) [14]. Several bacterial and fungal isolates have the capacity to directly increase plant biomass via growth hormone production and/or by providing plants with limiting macro- or micro-nutrients [13, 15–19]. Although diseases caused by pathogens have been shown to be directly or indirectly reduced by the addition of single or multiple beneficial microbes [4, 8, 20–23], how fungal root microbiota members with beneficial functions influence and are influenced by bacterial colonization remains less understood.

Sebacinales fungi (Basidiomycetes) are a remarkable group of plant mutualists with worldwide occurrence in soils and as endophytes. While individual Sebacinales strains can interact with roots in the absence of differentiated structures, they can also form specialized interactions with distinctive morphological characteristics on relevant hosts, as in orchid- or ectomycorrhiza symbioses [24]. Root colonization by these fungi improved host growth and development, increased grain yield, and enhanced root phosphate uptake in several plant species [25–28]. The positive effects of Sebacinales on the host plant extend well beyond growth and development and cannot be explained by enhanced host nutrition alone [24, 26, 29]. Recently, it was shown that effector molecules derived from the Sebacinales root endophyte *Serendipita indica* contribute to the establishment of the fungus−host interaction [30–33]. *S. indica* effectors suppress plant defence responses and modulate plant metabolism to promote compatibility in the roots, but their contribution to beneficial outcomes is unclear. Similarly, the nature of host transcriptional programs and signalling networks that lead to a mutually beneficial fungus−plant partnership are not well understood.

In the past few years, microbe−microbe interactions have emerged as an additional important element shaping plant host−microbe interactions [4, 22, 34, 35]. Using a soil-based split-root system, we demonstrated that both local and systemic colonization by the Sebacinales endophyte *Serendipita vermifera* (syn. *Sebacina vermifera*, hereafter *Sv*) afford protection against *Bs* infection and disease symptoms in *Hordeum vulgare* (barley) [4]. Here, we explore how *Sv* and *Bs* colonization capacities in two plant species, barley, and Arabidopsis, are modulated by the presence of individual members of the core bacterial microbiota or SynComs isolated from the barley rhizosphere [36] or Arabidopsis roots [37]. The finding that *Bs* also infects and causes disease symptoms in Arabidopsis roots motivated us to develop a set of physiological measurements to characterize disease severity and plant growth in Arabidopsis under different microbe treatment regimes. These measurements include ion leakage (quantified *via* electric conductivity) and photosynthetic activity (measured using pulse amplitude modulation fluorometry) as readouts for host cell death progression and biotic stress during the host−microbe interaction. Analyses of inter-kingdom activities in barley and Arabidopsis revealed that *Sv* can functionally replace the core bacterial component of the rhizosphere by mitigating pathogen infection and disease symptoms in both hosts. Additionally, we show that cooperation between bacteria and beneficial fungi leads to inter-kingdom synergistic beneficial effects, thereby providing insights into the complex relationships of the rhizosphere. Finally, RNA-seq experiments with selected bacterial strains alone or combined with *Sv* and/or *Bs* give insights to how microbes synergistically protect plants. We conclude that plants have evolved to preferentially accommodate communities that support their health and that root-associated prokaryotic and eukaryotic microbes can act synergistically with the plant host in limiting fungal disease.

## Material and methods

### Plant, fungal, and bacterial materials

Barley (*Hordeum vulgare* L. *cv* Golden Promise) and *Arabidopsis thaliana* Col-0 were used as plant hosts. *Serendipita vermifera* (MAFF305830) and *Bipolaris sorokiniana* (ND90Pr) were used as fungal models. The *At*SynCom consists of four bacterial strains from the *At*Sphere collection [37]. The *Hv*SynCom consists of 26 bacterial strains of an existing collection [36] as described in Fig. S1.

### Growth conditions and microbe inoculations

Barley seeds were surface sterilized in 6% sodium hypochloride for 1 h under continuous shaking and subsequently washed each 30 min for 4 h with sterile water. The seeds were germinated on wet filter paper in darkness at room temperature for 4 days, transferred to 1/10 PNM (Plant Nutrition Medium, pH 5.7) in sterile glass jars for growth at a day/night cycle of 16/8 h at 22/18 °C, 60% humidity under 108 µmol/m^2^s light intensity.

Arabidopsis seeds were surface-sterilized two times in 70 and 100% EtOH for 5 min each and sown on ½ MS (Murashige−Skoog-Medium including vitamins, pH 5.6) with 1% sucrose after ethanol removal. Following two days of stratification at 4 °C and darkness, the seeds were germinated at a day/night cycle of 8/16 h at 22/18 °C, 60% humidity, and a light intensity of 125 µmol/m^2^s for seven days. Seedlings of similar size were transferred to 1/10 PNM medium in 12 × 12 cm square Petri dishes 1 day prior to microbe inoculation.

Single bacterial strains were grown separately in liquid TSB medium (Sigma Aldrich) (15g/l) at 28 °C in darkness with shaking at 120 rpm for 1−3 days depending on growth rates. Final OD_600_ was adjusted to 0.01 prior to inoculation of single strains or mixtures in equal amounts for SynComs constitutions to a final OD_600_ of 0.01.

*Sv* was propagated on MYP medium [38] and *Bs* on modified CM [4] medium both containing 1.5% agar at 28 °C in darkness for 21 days and 14 days pre inoculation respectively. *Sv* mycelial and *Bs* conidia suspensions were prepared as described in [4].

Arabidopsis roots were inoculated either with *Sv* mycelium (1g/50ml), *Bs* conidia (5 × 10^3^ spores/ml), bacteria (OD_600_ = 0.01) or a mixture of organisms contained in 0.5ml sterile MilliQ water equally spread across individual plates. Barley roots were inoculated with 3ml of *Sv* mycelium (2g/50ml), *Bs* conidia (5 × 10^3^ spores/ml), bacteria (OD_600_ = 0.01) or a respective mixture of organisms per jar. Sterile MilliQ water was used as a control treatment. Arabidopsis and barley roots were harvested at 6 days post inoculation (dpi). Per biological replicate of each experiment and treatment, roots from 60 Arabidopsis plants or four barley plants were pooled. For RNA extraction roots of both plant species were washed thoroughly to remove extraradical fungal hyphae and epiphytic bacteria and snap-frozen in liquid nitrogen.

### Pulse-amplitude-modulation (PAM) fluorometry and ion leakage measurement

For PAM fluorometry and ion leakage assays, Arabidopsis plants were picked from the plate at 6 dpi. The plant roots were washed carefully and thoroughly to remove extraradical fungal hyphae and epiphytic bacteria and subsequently transferred to a 24 well plate containing 2 ml sterile MilliQ water per well. Five plants of the same treatment were transferred to each well. PAM fluorometry and ion leakage were measured every 24 h from 1 to 7 days post transfer (dpt) as described in [39] and as indicated in the figure legends.

### RNA isolation for RNA-seq and RT-PCR

RNA extraction for quantification of fungal endophytic colonization and RNA-seq, cDNA generation, and RT-PCR were performed as described previously [4]. The primers used are listed in Table S1.

### Statistical analyses

For fungal colonization and plant phenotypic analyses, as well as for quantification of disease symptoms and cell death, statistical evaluation was performed using either a one-way ANOVA and Tukey‘s post hoc test (*p* < 0.05) or a non-parametric Kruskal−Wallis test, followed by pairwise Mann–Whitney U-tests for multiple comparisons (FDR adjusted *p*-value < 0.05) depending on the experiment and readout as indicated in the figure legends.

### Genomic and transcriptomic data analysis

Stranded mRNA-seq Libraries were prepared according to the manufacturer’s instructions (Vazyme Biotech Co., Nanjing, China). Qualified libraries were sequenced on a HiSeq 3000 system instrument at Genomics & Transcriptomics Laboratory, Heinrich-Heine University, Germany (https://www.gtl.hhu.de/en.html) to generate 50 million reads with a 150 bp read length from two to three biological replicates. Reads with Illumina adaptors and sequence quality scores lower than 15 were removed using fastp [40]. High-quality sequencing reads were then aligned to the annotated reference genomes of the three organisms (barley: IBSC Morex v2, *Bipolaris sorokiniana*: Cocsa1, *Serendipita vermifera*: *Sebacina vermifera* MAFF 305830 v1.0, Table S2) using kallisto (v.0.46.1) [41]. Read count per transcript was converted into read count per gene using an in-house custom pipeline and R package tximport [42]. Potential batch effects were excluded with Combat-seq function in SVA package [43]. We selected 25,172 of 39,734, 10,178 of 12,250, and 13,376 of 15,312 genes having more than averaged five reads per condition for *H. vulgare*, *B. sorokiniana*, and *S. vermifera* respectively for the analysis (Tables S3, S4, and S5). The log2 fold difference of the gene expression between conditions was calculated with R package DESeq2 [44]. Genes with statistical significance were selected (FDR adjusted *p* value < 0.05). The consistency of normalized transcription from two to three biological replicates was confirmed by visualizing the distribution of read counts. Normalized read counts of the genes were also produced with DESeq2, which were subsequently log2 transformed. Functional annotation sets were combined using the Carbohydrate Active Enzyme database CAZy [45], the Gene Ontology GO Consortium [46], Kyoto Encyclopedia of Genes and Genomes KEGG [47], and EuKaryotic Orthologous Groups KOG [48], PFAM [49], Panther [50], MEROPS [51]. KOG, GO, KEGG, PFAM, Panther, MEROPS, best *O. sativa* hit homologues and best *A. thaliana* TAIR10 hit homologues were obtained from Phytozome, JGI (https://phytozome-next.jgi.doe.gov/). CAZymes, MEROPS, and GO terms were obtained based on KEGG, GO, and PFAM IDs using R packages KEGG.db, GO.db, and PFAM.db [52–54]. Fungal genomes and functional annotations were obtained from Mycocosm, Joint Genome Institute (https://mycocosm.jgi.doe.gov/mycocosm/home). The latest CAZy annotations were provided from CAZy team (www.cazy.org). Secreted proteins were predicted with Secretome pipeline described previously [55]. We identified the genes coding for CAZymes, lipases, proteases, small secreted proteins (less than 300 amino acids) as a subcategory. Fungal effectors were previously identified, which were combined with the predicted secretome information in this study [4]. We sorted significantly differentially regulated genes specific to the conditions (> 1 log2 FC; FDR adjusted *p* < 0.05) and visualized with R package UpSetR [56]. Such genes were grouped using K-means clustering with R package pheatmap [57]. Networks of k-means clustered genes were visualized with R package ggraph [58]. Genes expressed differently among the conditions were identified based on principal coordinates calculated with R package Vegan [59]. The first three principal coordinates were used to select high loading genes coding for glycosyl hydrolases and effectors of *B. sorokiniana*. Comparative analyses with a previous transcriptomic dataset [4] showed that 37 of the 50 top induced barley genes in response to *Bs* in soil are again detected to be significantly induced in the Barley_*Bs* vs Barley comparison in PNM (this study), indicating a large overlap of the highly responsive host genes to the pathogen in soil and PNM. Data are deposited at NCBI under the BioProject accession number: PRJNA715112.

### Gene co-expression analysis

A self-organizing map (SOM) was trained with the normalized read count of the selected replicates using Rsomoclu and kohonen [60, 61]. A total of 1015 nodes (35 × 29 matrix) was used with a rectangular shape (four neighbouring nodes). The resolution of 25 genes per node was applied for clustering, which was empirically optimized [62, 63]. The epoch of 1000 times more than the map size was applied (i.e., 1,015,000 iterations of learning, being 1015 map size times 1000). The genes showing similar regulation trends were grouped based on the mean transcription of the nodes. We examined genome-wide condition-specific transcriptomic patterns in graphical outputs (i.e., Tatami maps). Mean transcription values were calculated from the grouped genes per condition in each node (i.e., node-wise transcription). Then, using the node-wise transcription values, highly-regulated genes specific to each of the conditions were determined by fulfilling either of two criteria: 1) > 12.6 log2 reads (above 95th percentile of the entire transcribed genes); or 2) over ± 2 log2 transcriptional differences between testing conditions and a control. The process above was performed in a semi-automated manner using co-gene expression pipeline (SHIN+GO; [62–65]. R was used for operating the pipeline (http://www.R-project.org).

## Results

### Sebacinales associate with healthy Arabidopsis plants in diverse European locations

**Fig. 1:**
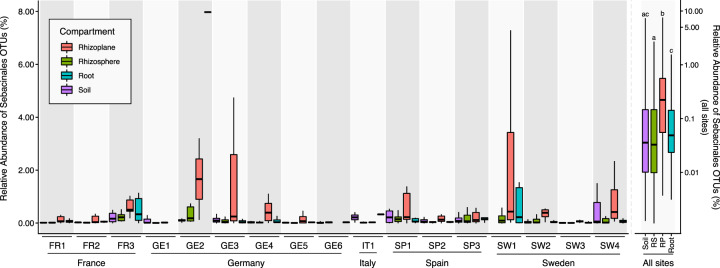
Abundance of Sebacinales in Arabidopsis roots of different European locations. Abundance and location of Sebacinales OTUs in nature suggest functional association of these fungi with *Arabidopsis thaliana*. Analysis of fungal (ITS1) OTUs belonging to the Sebacinales order from sequencing data obtained from samples of soil and root-associated microbial communities across 3 years and 17 European sites where naturally occurring *A. thaliana* populations were found [9]. A non-parametric Kruskal−Wallis test with a Dunn’s test for multiple comparisons on relative abundances of Sebacinales OTUs in different compartments, aggregated for all sites, shows that this fungal taxon is enriched in the rhizoplane compartment of *A. thaliana* roots compared to the other compartments. *Y*-axes have different scales for all sites (right scale) and single sites (left scale).

By monitoring root-associated microbial communities in natural *A. thaliana* populations, Thiergart et al. [9] showed that microbial community differentiation in the roots is explained primarily by location for filamentous eukaryotes and by soil origin for bacteria, whereas host genotype effects are marginal. We re-analyzed this dataset, including lower abundance operational taxonomic units (OTUs), and found that fungal OTUs of the order Sebacinales were significantly enriched in the rhizoplane compartment of healthy Arabidopsis plants in diverse European locations (Fig. 1). These environmental sampling data complement cytological studies which show that Sebacinales isolates colonize Arabidopsis by forming a loose hyphal mesh around roots with intracellular colonization limited to the root epidermis and cortex layer [38]. The frequent occurrence and enrichment patterns of Sebacinales OTUs in the roots of native Arabidopsis suggest a functional endophytic association with this host in nature. This finding motivated us to investigate the functional relevance and resilience of these fungi in a community context in the roots of Arabidopsis and to compare these with the beneficial effects observed in barley using bacterial synthetic communities.

### Protection mediated by *S. vermifera* and bacteria is synergistic and largely independent of the host

**Fig. 2:**
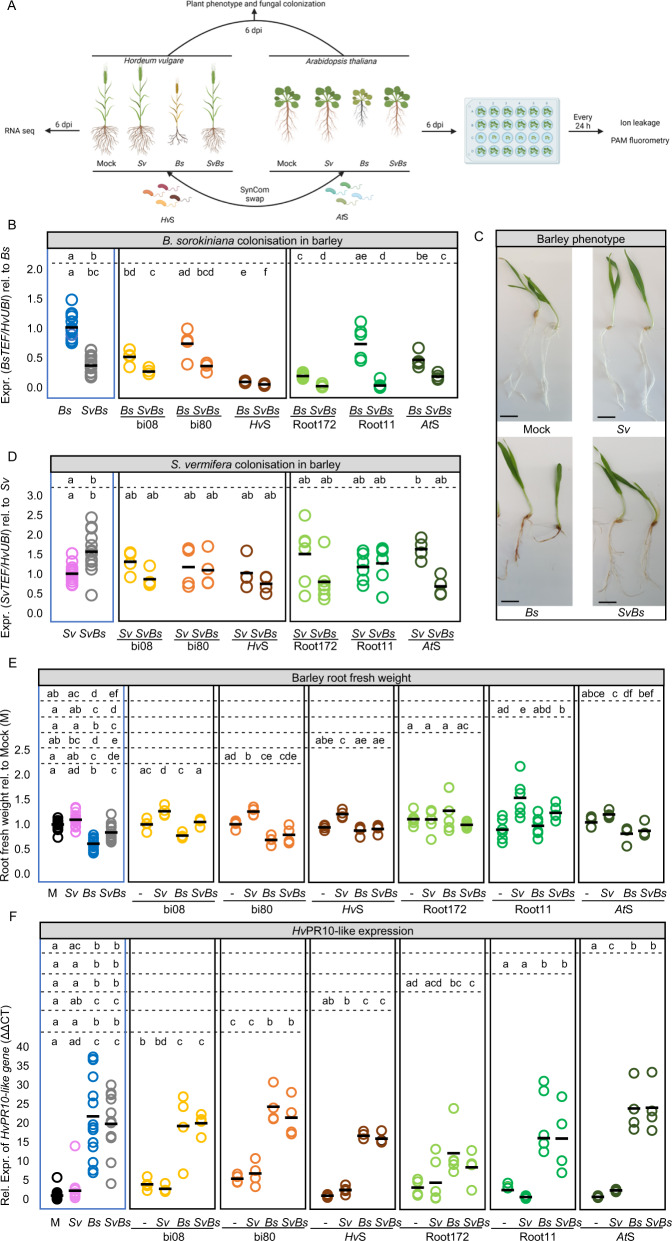
Barley root colonization and responses after fungal and/or bacterial inoculation at 6 dpi. *Sv* and the bacterial SynComs display beneficial effects on barley protection and plant growth. **A** Scheme depicting the experimental setup for barley and Arabidopsis. **B** *Bs* and **D** *Sv* colonization in barley roots. Fungal colonization was inferred by expression analysis of the fungal housekeeping gene *TEF* compared to the barley ubiquitin (*UBI*) gene (*n* = 4–14). **C** Pictures showing barley inoculated with water as a control (mock), *Sv*, *Bs,* or both fungi together, scale bar = 1 cm. **E** Barley root fresh weight per biological replicate normalized to mock inoculated plants (*n* = 4–14). **F** Relative expression of *HvPr10*-like gene (HORVU0Hr1G011720) to *UBI*. Different letters in the comparison between the tripartite panel (blue frame) and combinations of any other subpanel (defined by the dashed lines) represent statistically significant differences according to non-parametric Kruskal−Wallis test followed by pairwise Mann–Whitney U-tests for multiple comparisons (FDR adjusted *p*-value < 0.05).

We reported that *Sv* acts as an extended plant protection barrier in the rhizosphere, which reduces barley root infection and disease symptoms caused by the hemibiotrophic pathogen *Bs* on defined plant sugar-free minimal medium (PNM) and in natural soil [4]. Here we examined the role of *Sv* in augmenting the plant immune system in two different hosts for resilience against environmental threats in a bacterial community context by monitoring fungal colonization, plant growth promotion, protection, and transcriptional response (Fig. 2A).

**Fig. 3:**
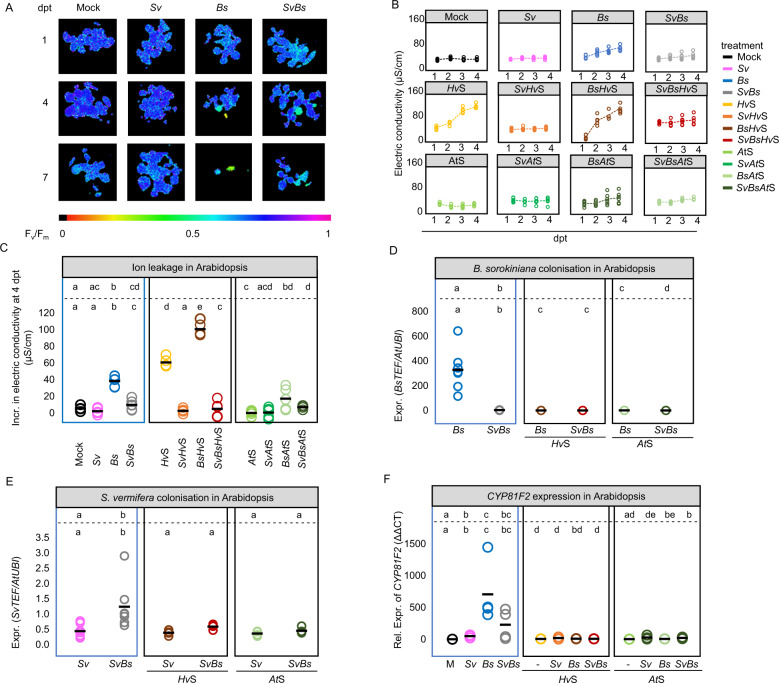
Arabidopsis root colonization and responses after fungal and/or bacterial inoculation. *Sv* and the bacterial SynComs display beneficial effects on Arabidopsis protection. **A** Arabidopsis photosynthetic activity (*F*_V_/*F*_M_) at 1, 4, and 7 days post transfer (dpt) corresponding to 7, 10, and 13 days post inoculation (dpi), after treatments with *Bs*, *Sv*, or both fungi together. Purple/dark blue, lighter colours, and black colour indicate high, reduced, and lack of PS II activity, respectively. **B** *Bs*- and *Hv*S-induced cell death measured via electric conductivity from 1 to 4 dpt (*n* = 6). **C** Total increase in electric conductivity from 1 to 4 dpt (*n* = 6). Statistical analyses were performed for each subpanel together with the tripartite panel (blue frame). **D** *Bs* and **E** *Sv* colonization in Arabidopsis inferred by expression analysis of the fungal housekeeping gene *TEF* compared with plant ubiquitin (*UBI*) (*n* = 4–7). **F** Expression of the Arabidopsis cytochrome P450 monooxygenase *CYP81F2* gene. Different letters in the comparison between the tripartite panel and combinations of any other panel (defined by the dashed lines) represent statistically significant differences according to non-parametric Kruskal−Wallis test followed by pairwise Mann–Whitney U-tests for multiple comparisons (FDR adjusted *p*-value < 0.05).

In the host barley, we confirmed the protective activity of *Sv* during *Bs* infection of root tissue (Fig. 2B, C) and additionally we observed enhanced *Sv* colonization through the presence of *Bs* at 6 dpi on PNM (Fig. 2D). In the host Arabidopsis, *Bs* infected seedlings displayed prominent disease symptoms at 6 dpi on PNM such as reduced main root length, rosette diameter, and lateral root number compared to mock-inoculated controls (Fig. S2A–C). *Bs* inoculated roots exhibited characteristic tissue browning, increased ion leakage, and a reduced photosynthetic active leaf area over time, indicative of host cell death progression (Fig. 3A–C and Figs. S2D,  S3). As shown for barley and in accordance with their growth rates in axenic cultures [4], *Bs* generated more endophytic biomass than *Sv* upon separate inoculations of Arabidopsis roots, determined by a quantitative reverse transcription PCR (RT-qPCR) test displaying the ratio between constitutively expressed single copy fungal (TEF) and plant (UBI) genes (Fig. 3D, E). Notably, *Bs* endophytic biomass and disease symptoms were substantially diminished in roots that were co‐colonized by *Sv* (Fig. 3A–D and Figs. S2A−C,  S3)*. Sv* endophytic colonization was enhanced by the presence of the pathogen also in this tripartite interaction (Fig. 3E). The enhanced *Sv* colonization in both hosts could be explained by the plant actively recruiting *Sv* to suppress the soil-born pathogen or *Sv* feeding on *Bs* and/or necrotic plant tissues.

Next, we determined whether bacterial strains isolated from the rhizosphere of barley (*Hv*SynCom) or the endosphere of Arabidopsis roots (*At*SynCom) can also protect barley and Arabidopsis from *Bs* infection. Both SynComs were able to reduce *Bs* colonization and partially rescue plant phenotypes caused by the pathogen in both hosts (Figs. 2B, E, 3B–D and Fig. S2B, C). Interestingly, the *Hv*SynCom alone, but not the *At*SynCom, caused increased ion leakage and reduced photosynthetic active leaf area in Arabidopsis (Fig. 3B, C and Fig. S3). This points towards an induction of host cell death in Arabidopsis by the non-native bacterial SynCom.

To clarify whether the observed host protection against *Bs* infection is a general property of root-associated bacterial strains or requires a community context, we inoculated functionally and taxonomically-paired bacterial strains from the *Hv-* and *At*SynComs (Fig. S1) individually or in combination with *Bs* on barley. We observed a strong reduction of the pathogen infection with the Proteobacteria strains bi08 (*Pseudomonas* sp.) and Root172 (*Mesorhizobium* sp.) but not with the Firmicutes strain bi80 (*Bacillus* sp.) and Root11 (*Bacillus* sp.) irrespective of the host species origin (Fig. 2B). This indicates that not all bacterial strains in the SynComs have the ability to protect the roots from *Bs* infection but the overall protection effect is maintained in a community context.

Next, we interrogated whether the observed beneficial effects on the plant hosts mediated by *Sv* or the bacterial strains are retained or altered during inter-kingdom interactions. For this, we co-inoculated barley and Arabidopsis roots with *Sv* and *Bs* in combination with a single bacterial strain or a SynCom. We found that *Sv* colonization was only marginally affected by the presence of the bacteria or positively affected in the case of the *At*SynCom in barley (Figs. 2D and 3E). The combined presence of *Sv* and bacterial strains led to a stabilized (reduced biological variation) or potentiated host protection against *Bs* infection (Figs. 2B and 3D). Potentiated protection to *Bs* infection was most evident during co-inoculation of *Sv* with Root11 in barley (Fig. 2B). These data show a robust inter-kingdom protective effect of *Sv* with bacteria against an invasive fungal root pathogen.

**Fig. 4:**
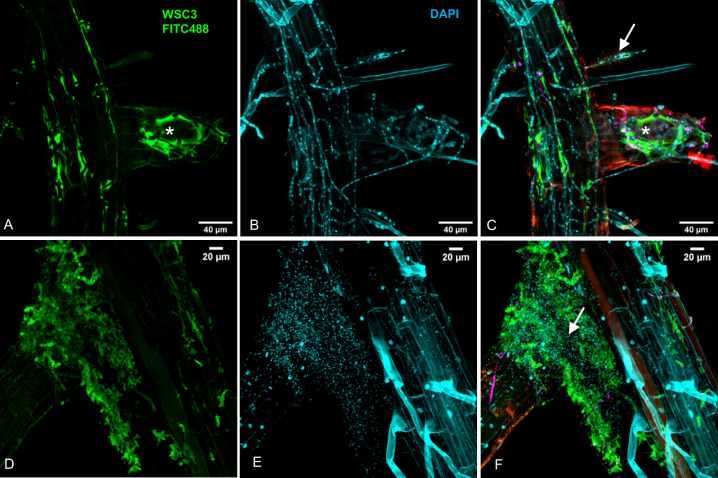
*Arabidopsis thaliana* Col-0 inoculated with *Bs* and Root172 at 7 dpi. Roots were fixed with 70% EtOH and stained with the β-1,3-glucan binding lectin WSC3-FITC488, which binds to the fungal matrix (in **A** and **D**), and the fluorescent DNA stain DAPI (in **B** and **E**). Overlays (in **C** and **F**). Confocal images were recorded using a Leica TCS-SP8 confocal microscope. White arrows: lysis of *Bs* matrix in the presence of Root172. Asterisks: intact fungal matrix.

Finally, to measure whether the host plant contributes to the effects displayed by *Sv* and the examined bacterial strains in limiting pathogen biomass, we additionally performed direct microbe−microbe confrontation assays on PNM. In these assays, we largely recapitulated the antagonism observed against *Bs in planta* with a general reduction of *Bs* colony areas in the presence of bacteria and/or *Sv* but not with Root11 alone (Fig. S4). We, therefore, concluded that microbe−microbe interactions rather than the host plant are most important for conferring the root protective properties of *Sv* or the tested bacteria. This notion is also supported by *in planta* cytological analyses in which we observed a direct interaction between *Bs* and Root172 at the rhizoplane of Arabidopsis and extensive lysis of the fungal extracellular polysaccharide matrix surrounding *Bs* hyphae (Fig. 4).

### *S. vermifera* confers plant growth promotion in cooperation with selected root-associated bacteria

*Sv* promotes plant growth in different host species at late stages of colonization [66–68]. At an early colonization time point of 6 dpi in barley, neither *Sv* alone nor any of the single bacterial strains or SynComs led to a significant change in root fresh weight (Fig. 2E). By contrast, a combination of *Sv* and bacterial strains Root11, bi08 or bi80, significantly increased barley root fresh weight at 6 dpi (Fig. 2E). This early inter-kingdom mediated root growth promotion effect was strain-specific, not restricted to bacterial strains isolated from the barley rhizosphere, and maintained in a community context (Fig. 2E). Co-inoculation with heat-inactivated bacterial SynComs failed to increase barley root fresh weight (Fig. S5A), underlining the importance of living bacteria in promoting root growth.

In Arabidopsis, we observed root growth inhibition at 6 dpi upon inoculation with *Bs* or the SynComs irrespective of the number of bacterial strains and their host origin (Fig. S2A). Co-inoculation with *Sv* largely alleviated the *Bs*-mediated root growth inhibition but did not increase root or shoot size compared to controls (Fig. S2A–C). Only the combination of Root172 with *Sv* led to a significant increase in Arabidopsis rosette diameter at 6 dpi (Fig. S2E, F). This phenotype was, however, not retained in a bacterial community context, suggesting that it is less robust and/or plant growth-promoting microbes suffer from competition by other community members.

### Inter-kingdom synergistic beneficial activities are not associated with extensive host transcriptional responses

**Fig. 5:**
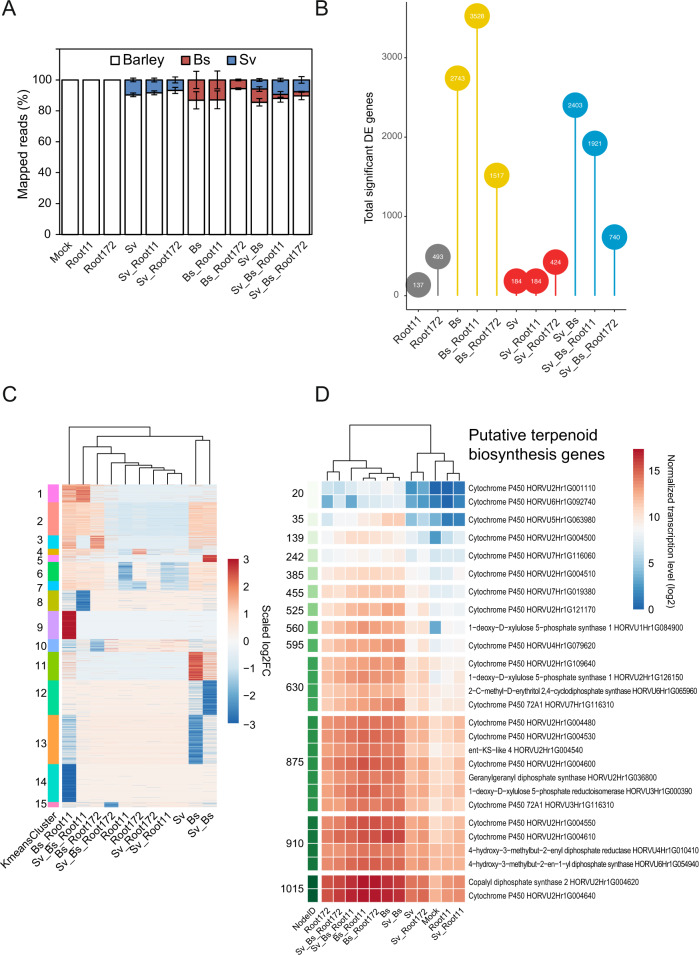
Analysis of barley root transcriptional responses to fungal and bacterial colonization at 6 dpi. **A** Proportion of reads mapped to the organisms per sample ± SEM. A total of 34 RNA-seq samples were mapped to the corresponding organisms. Mock: *Hordeum vulgare*. See Table S2. **B** Total number of differentially expressed genes per condition (> 1 log2FC; FDR adjusted *p*-value < 0.05) in comparison to barley mock control. The numbers are shown in the circles. See Fig. S8 and Table S6. **C** K-means clustering of differentially expressed genes grouped into 15 clusters. A total of 5,539 differentially expressed genes are used for (**B** and **C**). See Tables S6 and S7. **D** Normalized transcription level of genes putatively involved in terpenoid phytoalexin synthesis. Averaged transcription in log2 is shown per condition. Terpenoid phytoalexin synthesis pathway in barley was published earlier [4]. See Table S8. *Bs: Bipolaris sorokiniana. Sv: Serendipita vermifera*. Root11 and Root172: *A. thaliana* root-associated bacterial strains Root11 and Root172 from the *At*Sphere collection.

To investigate mechanisms underlying the synergistic beneficial effects displayed by a combined fungal endophyte and bacterial inoculation, we analyzed the barley root transcriptome during fungal and bacterial colonization by RNA‐seq. The multipartite systems used for transcriptomics included the two fungi (*Sv* and *Bs*) and the bacterial strains Root172 and Root11, selected based on their distinctive and robust *in planta* activities with *Bs* and *Sv* at 6 dpi. Namely, Root172 conferred strong host protection against *Bs* whereas Root11 had a strong root growth promotion phenotype (Fig. 2B, E). To determine species representation in the Illumina RNA-seq reads, we mapped reads to annotated genes of the barley and fungal reference genomes. Bacterial reads were not present in the dataset due to the method used for the library preparation. On average, 7.9% of reads matched *Sv* genes in all endophyte‐containing samples (Fig. 5A; Table S2). By contrast, the relative abundance of reads mapping to *Bs* genes decreased from 13.1% (*Bs* alone) to 8.6%, 12.9% or 5.7% when *Sv*, Root11 or Root172 were co‐inoculated with the fungal pathogen, respectively. Co-inoculation of Root11 or Root172 with *Sv* and *Bs* reduced the relative abundance of pathogen reads, to 2.6 and 2.7%, respectively (Fig. 5A; Table S2). The reduction in *Bs* reads with *Sv* and/or bacterial strains likely reflects reduced *Bs* biomass, confirming the quantitative RT‐PCR analysis (Fig. 2B). To dissect barley transcriptomic trends and identify differentially expressed genes (DEG), we examined genes that were induced or repressed under specific conditions after transcript mapping and quality assessment (Fig. S6, see “Methods”). Consistent with our previous data [4], we detected only a weak host transcriptomic response to *Sv* (184 DEG with log2FC>1, Fig. 5B, Fig. S7; Table S6). Neither presence of the bacterial strains nor combined presence of bacteria and *Sv* led to an extensive host transcriptional response (Fig. 5B, Fig. S7; Table S6). Thus, the observed early root growth-promoting effects mediated by *Sv* with Root11 in barley were not accompanied by a strong host transcriptional response (with 14 DEG specific to this condition—3 up and 11 downregulated genes—Fig. S7B, C; Table S6).

Conversely, infection with *Bs* resulted in 2,743 barley DEG. Co-inoculation of *Bs* and Root172 reduced barley DEG to 1,517, whereas Root11 with *Bs* produced a larger number of DEG (3,528) compared to *Bs* alone (Fig. 5B and Fig. S7). Grouping DEG according to expression patterns identified 15 clusters of highly up or downregulated barley genes specific to one or more condition/s and showed that the barley response to co-inoculation with *Bs* and Root11 was most different from all other conditions (Fig. 5C; Table S7). To identify functional categories in co-regulated genes, we employed a SOM to group genes into nodes displaying similar regulation (Fig. S8; Table S3) and we performed GO enrichment analyses (Fig. S9). These analyses showed that *Bs* alone strongly induced a barley immune response and terpenoid phytoalexin production (Fig. 5D; Table S8). Root11 had no effect on immunity or terpenoid phytoalexin production, whereas Root172 slightly induced an immune response. Notably, co-inoculation of Root11 with *Bs* provoked a higher activation of immunity genes and repression of host cell wall biosynthesis and DNA modification compared to the pathogen alone (Fig. S9).

In accordance with the reduction of *Bs* biomass and disease symptoms, the presence of *Sv* reduced the number of barley DEG in response to *Bs* (Sv_Bs: 2,403; Fig. 5B). This reduction was most pronounced in combination with the bacterial strains, especially with Root172 which had the strongest effect on *Bs* colonization (Sv_Bs_Root11: 1,921; Sv_Bs_Root172: 740; Fig. 5B, Fig. S7; Table S6). Consistently, the expression of barley genes associated with terpenoid phytoalexin production was partially reduced in the multipartite interactions compared to *Bs* alone (Fig. 5D). The barley root gene expression data shows that the cooperative action of *Sv* with bacteria protects barley roots from *Bs* infection without extensive host transcriptional mobilization of immunity and defence metabolic pathways.

To test the above observation further, we investigated the immune-modulatory proprieties of the beneficial *Sv* fungal and bacterial strains in roots of Arabidopsis and barley by using specific marker genes. In Arabidopsis, we observed a reduction of the expression of the gene encoding for the cytochrome P450 monooxygenase CYP81F2 involved in indole glucosinolate biosynthesis and defence [69] in *Bs* infected roots co-inoculated with *Sv* and/or the bacteria compared to *Bs* alone (Fig. 3F) which correlates well with the reduced pathogen load. In barley, we previously identified a *PR10* family gene (HORVU0Hr1G011720, hereafter referred to as *HvPR10*‐like) as a robust marker for induced immune responses to *Bs* colonization [4]. RNA-seq and quantitative RT‐PCR analyses confirmed that *HvPR10*‐like expression was highly induced by *Bs* infection of barley roots. By contrast, *HvPR10*‐like expression was weakly induced by *Sv* and/or the bacterial strains (Fig. 2F). Despite the strong reduction in pathogen infection and disease symptoms upon co-inoculation with *Sv* and bacteria, we found that *Bs*-induced *HvPR10-like* expression was generally maintained in all combinations (Fig. 2F). This result indicates that *HvPR10-like* expression is driven principally by the pathogen and impacted less by the presence of *Sv* and bacteria. Only co-inoculation of Root172 and *Sv*, which displayed the strongest protection against *Bs* infection, significantly lowered *Bs*-induced *HvPR10-like* gene expression (Fig. 2F). Hence, in conclusion, despite the general decreased barley transcriptional response to *Bs* and the lower pathogen load, the activation of specific immune responses such as the *HvPR10-like* gene were still in place in the presence of *Sv* and/or bacteria in this host.

### Synergistic actions of *S. vermifera* and bacteria reduce the virulence potential of endophytic *B. sorokiniana*

**Fig. 6:**
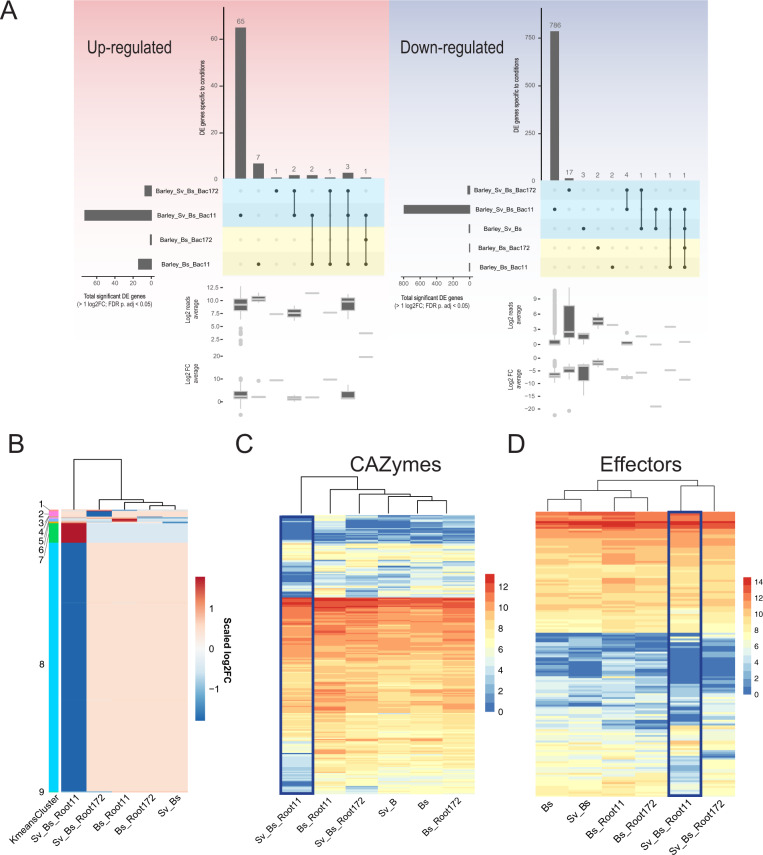
*B. sorokinana* transcriptional responses to *S. vermifera* and bacteria during infection of barley at 6 dpi. **A** Condition-specific differentially expressed *B. sorokiniana* genes (> 1 log2FC; FDR adjusted *p*-value < 0.05) compared to barley infection alone. Horizontal bars: total number of DEG per condition. Vertical bars: number of genes unique/shared for intersections. See Table S6. **B** K-means clustering of 923 differentially expressed genes grouped into nine clusters. See Table S7. **C** Averaged log2 read counts of predicted secreted CAZyme coding genes. **D** Averaged log2 read count of predicted effector coding genes. See Table S9. *Bs*: *Bipolaris sorokiniana*. *Sv*: *Serendipita vermifera*. Root11 and Root172: *A. thaliana* root-associated bacterial strains Root11 and Root172.

To examine mechanisms underlying the cooperative antagonistic behaviour of *Sv* and the bacteria towards *Bs*, we analyzed the fungal transcriptomes during barley root colonization at 6 dpi. We previously reported that fungal transcriptome changes are driven mainly by their interactions with the host and that *Sv* effects on the *Bs* transcriptome occur mostly in the rhizosphere [4]. Consistent with this notion, *Sv* or the bacterial treatments alone had little impact on the transcriptome of endophytic *Bs*. By contrast, the combined presence of *Sv* and Root11 had a strong impact on the *Bs* transcriptome with 65 up- and 786 downregulated genes (Fig. 6A; Table S6). DEG of *Bs* during root infection were grouped into nine clusters (Fig. 6B; Table S7). The largest *Bs* cluster (#8) contained genes that were repressed compared to *Bs* infection of barley alone. Among the top ten repressed genes in this cluster there were 4 *Bs* genes encoding for glycoside hydrolases (Table S7). This prompted us to look into the expression of all *Bs* CAZyme and effector genes.

**Fig. 7:**
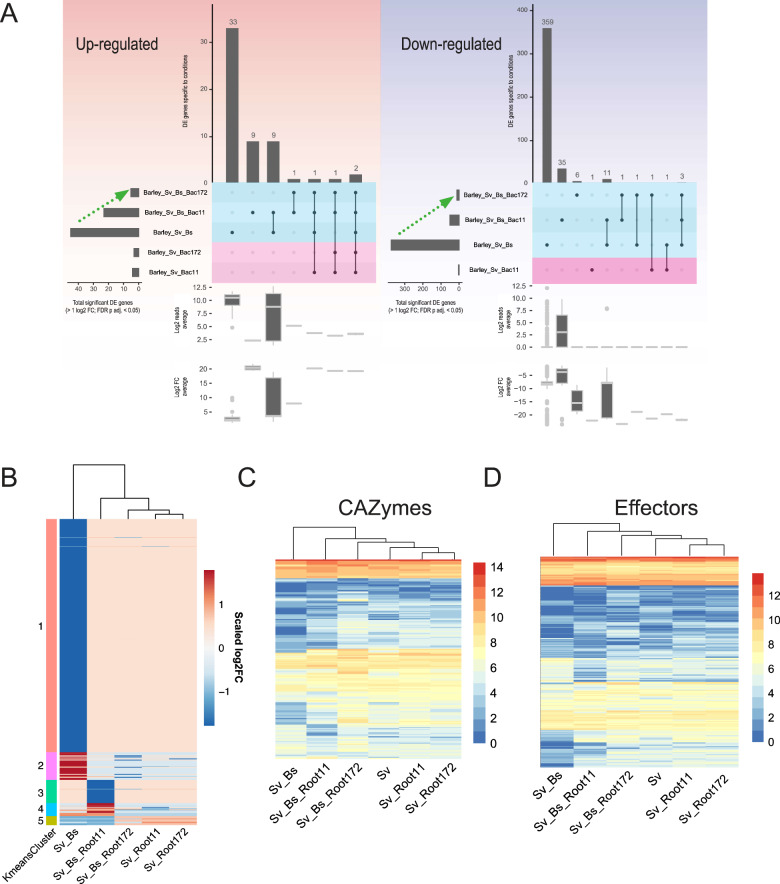
*S. vermifera* transcriptional responses to *B. sorokinana* and bacteria during colonization of barley at 6 dpi. **A** Condition-specific of differentially expressed genes (> 1 log2FC; FDR adjusted *p*-value < 0.05) are identified by comparing to the control condition (i.e., fungus alone). Horizontal bars: total number of DEG per condition. Vertical bars: number of genes unique/shared for intersections. See Table S6. **B** K-means clustering of 520 differentially expressed genes. See Table S7. **C** Averaged log2 read count of predicted secreted CAZyme coding genes. **D** Averaged log2 read count of effector coding genes. See Table S9. *Bs*: *Bipolaris sorokiniana*. *Sv*: *Serendipita vermifera*. Root11 and Root172: *A. thaliana* root-associated bacterial strains Root11 and Root172.

We observed a general transcriptional repression for genes in these categories by the combined presence of *Sv* and Root11, possibly explaining the reduced *Bs* colonization of roots (Fig. 6C, D, Figs. S10,  S11; Table S9). Notably, *Bs* gene cluster #7 (with genes specifically induced in the combined presence of *Sv*_*Bs*_Root11) contained six upregulated genes potentially participating in the production of antibacterial compounds related to chrysoxanthone, neosartorin, and emodin (Fig. S12; Tables S10, S11, and S12) [70–72]. Hence, it is possible that *Bs* actively engages in antagonizing Root11 in the presence of *Sv* at 6 dpi. On the other hand, upon *Bs* co-inoculation with Root11 we observed induced expression of fungal effector and CAZyme genes (cluster 5 in Fig. 6B,  C, D and Figs. S10,  S11) such as several AA9, GH43, CE5, PL1, and PL3 that are known to be enriched in plant-associated fungi [38, 73]. This observation might explain the increased host immune response to the combined presence of *Bs* and Root11. Transcriptional changes in endophytic *Sv* in response to the other microbes in barley roots were generally smaller and predominantly driven by *Bs* pathogen load and the associated barley immune response (Fig. 7, Figs. S13,  S14; Tables S6,  S8,  S9). This is in agreement with our previous data, which suggests that *Sv* transcriptional response is likely driven by the changes in the plant host environment due to the pathogen activity rather than by direct interaction with *Bs* inside the root [4].

## Discussion

In complex environments, plant−microbe interactions are not only shaped by the plant immune system [20, 74, 75] but also by microbe−microbe competition and co-operation, acting directly on or as an extension to plant immunity [76, 77]. Recent studies reveal the importance of root-associated bacteria for plant survival and protection against fungi and oomycetes [8, 78–81]. Much less attention has been paid to the role of widely distributed beneficial endophytic fungi in a multi-kingdom context. Here we show that the effects on host growth and protection that are conferred by the Sebacinales member *S. vermifera* in bipartite and tripartite interactions [4, 82] are retained in a community context. The observed robust protective function and stability of *Sv* colonization is likely due to its ability to adapt to changes in the plant host environment [4]. The strength of its protection against an aggressive root fungal pathogen (*Bs*) is underscored by the observation that *Sv* can functionally replace core bacterial microbiota members in mitigating pathogen infection and disease symptoms in distantly related plant hosts. This finding is in accordance with Arabidopsis root microbiota samplings across European habitats which shows Sebacinales fungi to be of low abundance but consistently present in the host roots and the rhizosphere. Our data highlight the potential importance of widespread root fungal endophytes in maintaining plant host physiological fitness in nature, thereby emphasizing that low-abundance microbes can play a significant role in microbiota beneficial functions and should be considered when designing SynComs with multiple traits, such as resilience and protective activities.

Strikingly, the presence of *Sv* additionally stabilizes and potentiates the protective activities of root-associated bacteria and mitigates the negative effects caused by the non-native *Hv*SynCom in Arabidopsis (Fig. 3B–D and Fig. S3), revealing a more general protective activity of root endophytic fungi. The induction of cell death by the barley-derived SynCom in Arabidopsis could be due to the presence of specific bacterial strains that are absent in the *At*SynCom. One such bacterial group that is well represented in the *Hv*SynCom but absent in the *At*SynCom used in this study is the Pseudomonadales. Several members of this group are reported to be pathogenic whereas others with very few genome differences promote plant growth and exert biocontrol activities against different fungal pathogens [83]. However, we did not observe an increase in ion leakage upon inoculation with the *Pseudomonas* strain bi08 or other members of the *Hv*SynCom when inoculated alone (Fig. S5B–E). The pathogenicity of a single bacterial strain is likely to be suppressed in a community context, as observed for *Bs* (Figs. 2, 3). Thus, another explanation to the negative effects of the *Hv*SynCom in Arabidopsis but not in barley might be a lack of adaptation to Arabidopsis. This notion is supported by a recent analysis that detected a clear signature of host preferences among commensal bacteria from diverse taxonomic groups, including Pseudomonadales in Arabidopsis and *Lotus japonicus* [84].

Our transcriptomic analyses show that the effects of the tested bacterial strains in tripartite associations differ substantially. The general decreased barley transcriptional response to the pathogen driven by the Rhizobiales strain Root172 (Fig. 5B) and the lysis of the fungal matrix at the host rhizoplane (Fig. 4) suggest that this bacterial strain most likely acts directly on *Bs*. This is also supported by the strong antagonism of *Bs* growth in confrontation assays irrespective of the presence of a host plant (Fig. 2B and Fig. S4). Taken together, these results point to Root172 as a possible biocontrol agent against *Bs* and potentially other root-infecting pathogens. The impact of Root172 contrasted strikingly with that of the Bacillales strain Root11, which did not limit *Bs* growth but rather enhanced *Bs* pathogenicity in barley. Notably, combining these two bacterial strains with *Sv* led to a restriction of *Bs* that exceeded the protective benefits of *Sv* and the bacteria alone (Fig. 2B). These synergistic beneficial effects are decoupled from extensive host transcriptional reprogramming (Fig. 5B) and cannot be solely explained by enhanced *Sv* growth (Fig. 2D and Fig. S4B) as speculated for other fungal-bacterial synergistic beneficial effects [85, 86]. Our transcriptional and phenotypic data further suggest that *Sv*—bacterial synergism in protecting host roots have also a component that is additive because the underlying antagonistic mechanisms displayed by the fungal root endophyte and the bacterial strains are likely to be distinct and explained mainly by direct microbe−microbe interactions outside the plant. Nonetheless, we have observed a higher level of inter-kingdom mediated antagonism on *Bs* in presence of the host (Fig. 2B and Fig. S4). This suggests a minor but relevant host-dependent effect that needs to be addressed.

At the early time point of 6 dpi, growth promotion was only observed in the combined presence of *Sv* and certain bacterial strains with the strongest effect during co-inoculation with Root11 in barley and Root172 in Arabidopsis (Fig. 2E and Fig. S2E). Furthermore, growth promotion required living microbes, as co-inoculation with heat-inactivated bacteria did not increase the root fresh weight in barley. Commensal bacteria in the rhizosphere can trigger plant growth promotion and resistance to pathogen [20, 21, 87]. Among them, strains belonging to the genus *Bacillus* are often used as bioagents due to their function in eliciting ISR (induced systemic resistance) as well as growth promotion [21, 88]. However, plant growth-promoting bacteria (PGB) and Sebacinales mediated growth promotion are often reported during later stages of colonization. The early host growth enhancement observed with *Sv* and the bacteria might thus confer a competitive advantage for plants in nature. It is striking that the growth-promoting effect is not accompanied by an extensive host transcriptional response with only 14 barley DEG being specific to this condition (Table S6). Interestingly, several of these genes display differential expression across barley accessions (analyzed using Genevestigator) compared to the cultivar Golden Promise. It would therefore be informative to test growth outcomes of combined *Sv* and e.g., Root11 inoculation in different barley varieties/ecotypes. The resulting synergistic inter-kingdom benefits in plant protection against fungal disease and in plant physiology are in line with studies of the Sebacinales fungus *S. indica* with single bacterial strains on tomato [85, 89, 90], rice [91], barley [92], and chickpea [93] and underline the broad functional relevance in plant health for fungi of the order Sebacinales in multi-kingdom environments. Inter-kingdom benefits in plant–beneficial microbe interactions were reported also for native isolates of *Trichoderma* spp. and *P. fluorescens* against *Ralstonia* spp. in tomato and with *B. velezensis* against *Fusarium* in gooseberry [23, 94], suggesting that the combined application of beneficial fungi and bacteria has strong potential as biocontrol agents.

The deployment of microbiota as biocontrol agents for crop protection and enhancement is an ancient concept that is gaining increased relevance in modern agriculture [95–97]. Plant protection and growth promotion properties conferred by microbial consortia have been found to be more resilient than the use of single strains [95]. Moreover, Duran et al. 2018 showed that a complex SynCom consisting of bacteria, fungi, and Oomycetes led to the strongest beneficial effects on Arabidopsis growth and survival compared to mono-kingdom or small SynCom associations and hypothesized that selective pressures over evolutionary time favour inter-kingdom microbe−microbe interactions over interactions with single microbial strains [8]. Inter-kingom associations are frequently observed between members of the Sebacinales and bacteria. Different Sebacinales species host endobacteria of the orders *Bacillales* (genera *Paenibacillus*), *Pseudomonadales* (*Acinetobacter*) and *Actinomycetales* (*Rhodococcus*) and its close relative *S. indica* hosts an endobacteria of the order *Rhizobiales* (*Rhizobium radibacter*) [98]. Beneficial effects of these intimate inter-kingdom interactions on the plant host and the fungus itself were described between *S. indica* and *R. radibacter* [98, 99] and for interactions between arbuscular mycorrhizal fungi and bacteria belonging to different species of the orders Proteobacteria (*Rhizobiales*) and Firmicutes (*Bacillales*) [100]. Considering the pervasiveness of beneficial effects conferred by *Sebacinales* and bacteria compared to the vulnerability of *Bs* in a multipartite context, our data support the hypothesis that the establishment of beneficial inter-kingdom interactions in the plant microbiota is an evolutionary conserved and robust trait.

## Supplementary information

Supplemental Figures

Table S1

Table S2

Table S3

Table S4

Table S5

Table S6

Table S7

Table S8

Table S9

Table S10

Table S11

Table S12
