# Supplementary material for: The fungal root endophyte Serendipita vermifera displays inter-kingdom synergistic beneficial effects with the microbiota in Arabidopsis thaliana and barley
Source: ISME J. 2021 Oct 22;16(3):876–89. doi: 10.1038/s41396-021-01138-y (PMC8857181; doi:10.1038/s41396-021-01138-y)
Supplement: Supplementary file 1 — Supplemental Figures [file 41396_2021_1138_MOESM1_ESM.pdf]

Supplementary material and methods and figures

The fungal root endophyte *Serendipita vermifera* displays inter-kingdom synergistic beneficial effects with the microbiota in *Arabidopsis thaliana* and barley

Lisa K. Mahdi[1*], Shingo Miyauchi[2*], Charles Uhlmann[2], Ruben Garrido-Oter[2,3], Gregor Langen[1], Stephan Wawra[1,3], Yulong Niu[1,2], Senga Robertson-Albertyn[4], Davide Bulgarelli[4], Jane E. Parker[2,3] and Alga Zuccaro[1,3#]

[1] University of Cologne, Institute for Plant Sciences, Cologne, Germany
[2] Max Planck Institute for Plant Breeding Research, Department of Plant Microbe Interactions, Cologne, Germany
[3] Cluster of Excellence on Plant Sciences (CEPLAS)
[4] University of Dundee, Plant Sciences, School of Life Sciences, Dundee, United Kingdom

[*] These authors contributed equally
[#]corresponding author: azuccaro@uni-koeln.de

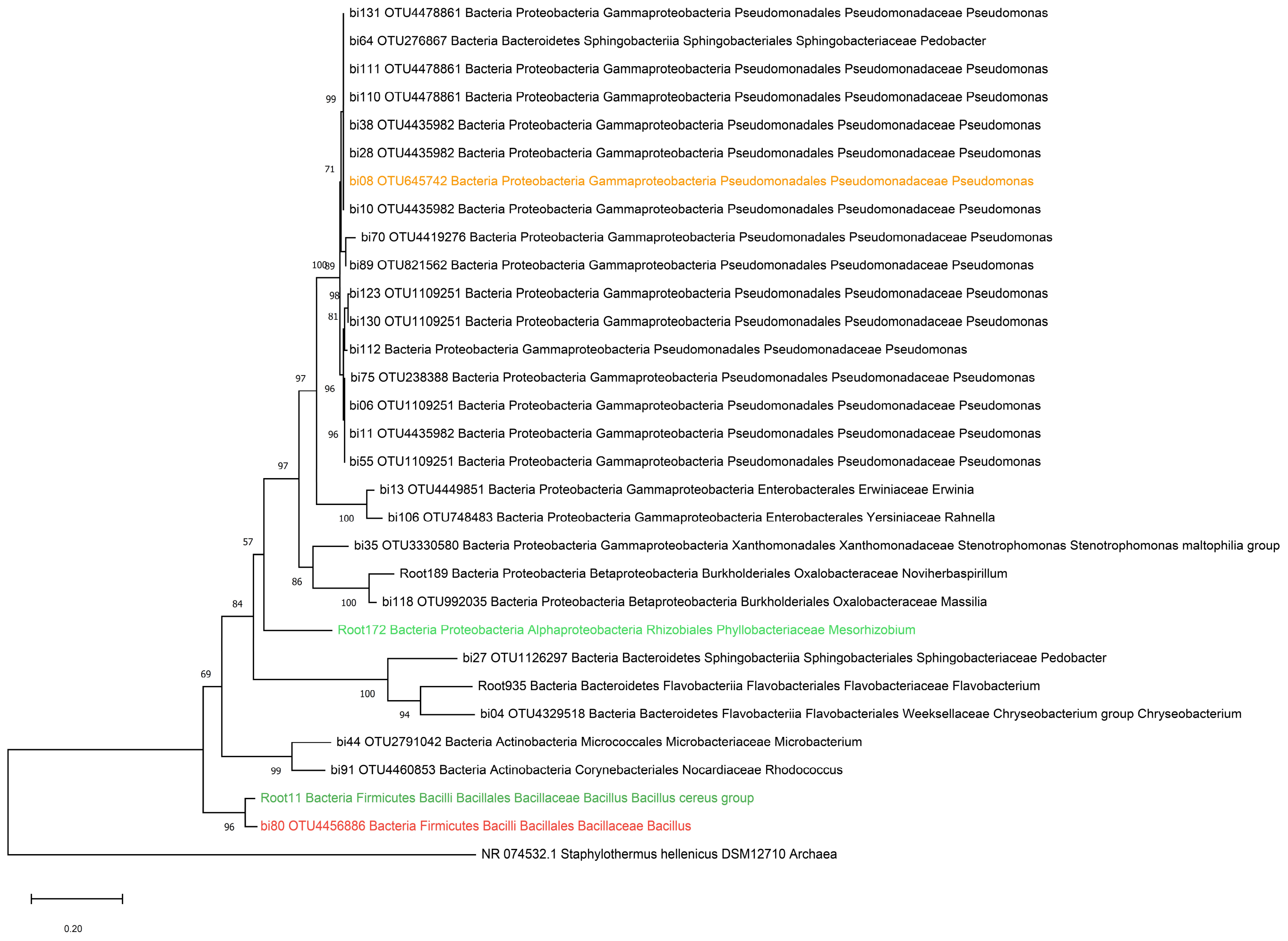

Fig. S1: Phylogenetic tree of Arabidopsis and barley associated bacteria. The evolutionary history was inferred from 16S rRNA genes by using the Maximum Likelihood method and Tamura-Nei model (Tamura *et al*., 1993). The percentage of trees in which the associated taxa clustered together is shown next to the branches. The tree is drawn to scale, with branch lengths measured in the number of substitutions per site. Evolutionary analyses were conducted in MEGA X (Kumar *et al*., 2018). Taxonomy of strains was inferred by blast searches against NCBI rRNA/ITS databases.

Kumar S., Stecher G., Li M., Knyaz C., and Tamura K. (2018). MEGA X: Molecular Evolutionary Genetics Analysis across computing platforms. *Molecular Biology and Evolution* 35:1547-1549

Tamura K. and Nei M. (1993). Estimation of the number of nucleotide substitutions in the control region of mitochondrial DNA in humans and chimpanzees. *Molecular Biology and Evolution* 10:512-526.

.

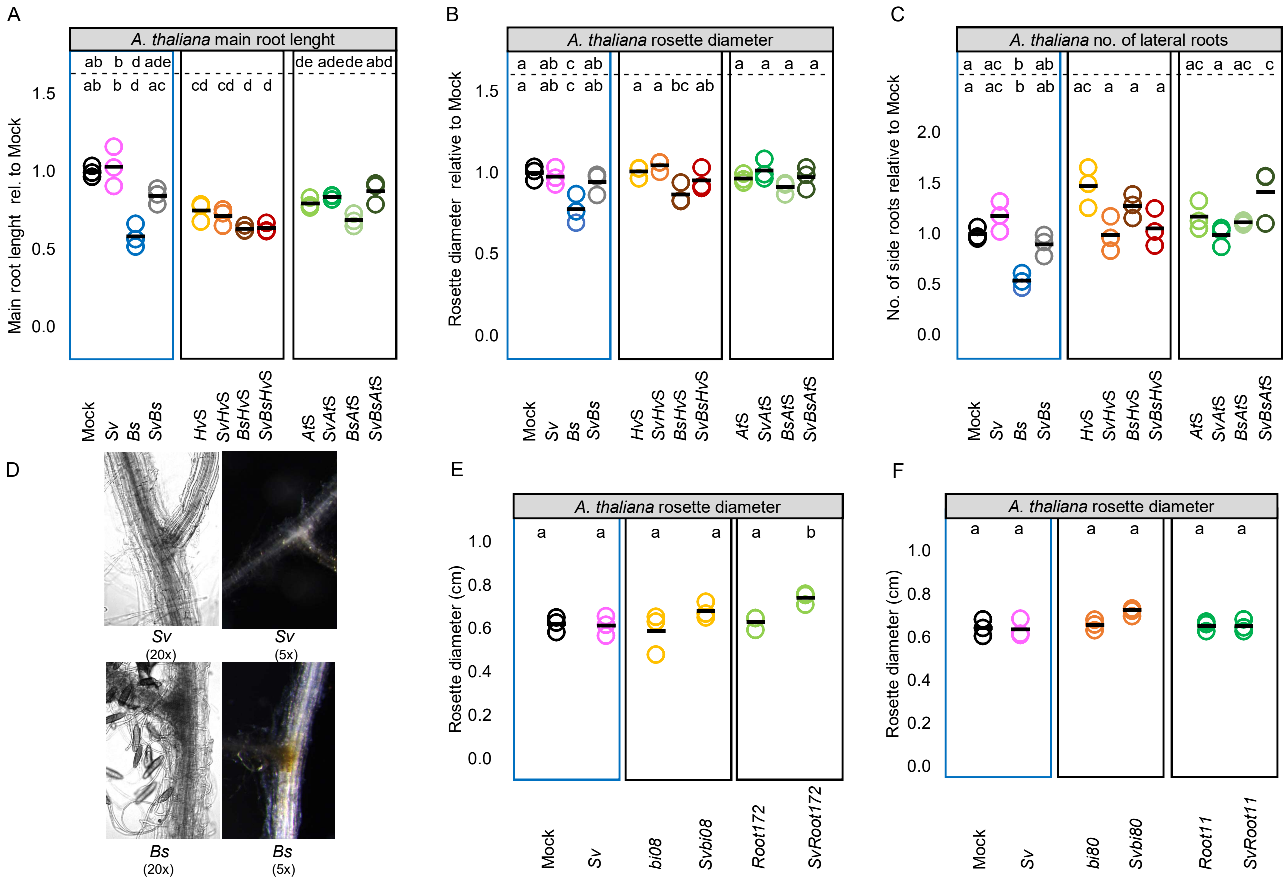

Fig. S2: Phenotypic analysis of Arabidopsis roots with *Sv* and/or *Bs* with or without the bacterial SynComs or single bacterial strains. A) The main root length, B) the rosette diameter and C) the number of lateral roots of Arabidopsis seedlings that were inoculated in dipartite, tripartite and multipartite systems with *B. sorokiniana* (Bs), *S. vermifera* (Sv) and the bacterial synthetic communities *Hv* SynCom (*Hv*S) or *At* SynCom (*At*S) relative to control plants (mock) at 6 dpi (n = 3, 60 plants per replicate). D) Pictures of *Sv* and *Bs* inoculated Arabidopsis roots at 6 dpi in 5x and 20x magnification. E) *A. thaliana* rosette diameter in presence or absence of *Sv* and the Proteobacteria strains bi08 or Root172 (n = 3, 60 plants per replicate). F) *A. thaliana* rosette diameter in presence or absence of *Sv* and the Firmicutes strains bi80 or Root11 (n = 3, 60 plants per replicate). Different letters represent statistically significant differences according to one-way ANOVA and Tukey‘s post-hoc test ($p < 0.05$).

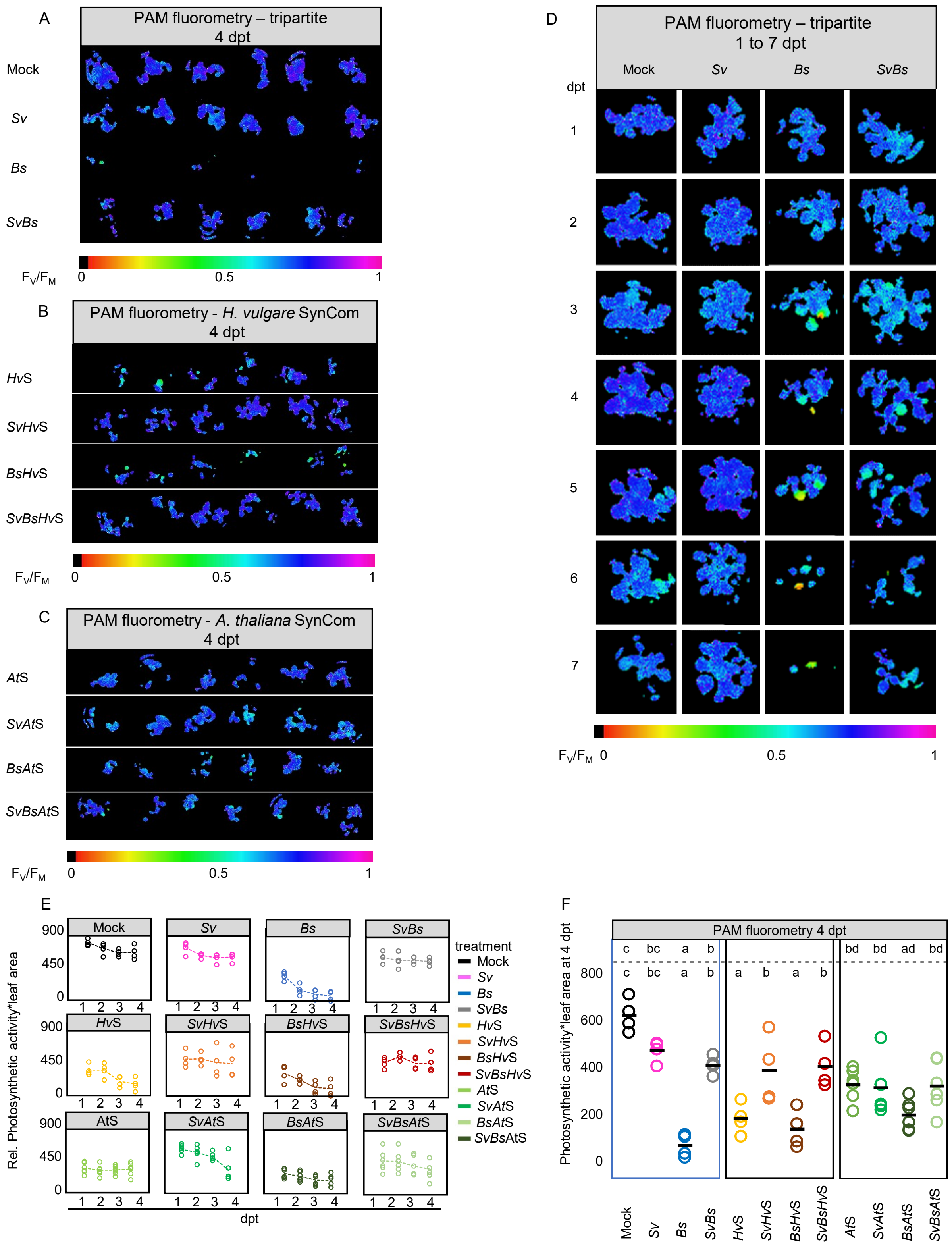
A
PAM fluorometry – tripartite
4 dpt
Mock
Sv
Bs
SvBs
FV/FM 0 0.5 1
B
PAM fluorometry - H. vulgare SynCom
4 dpt
HvS
SvHvS
BsHvS
SvBsHvS
C
PAM fluorometry - A. thaliana SynCom
4 dpt
AtS
SvAtS
BsAtS
SvBsAtS
D
PAM fluorometry – tripartite
1 to 7 dpt
dpt
E
Rel. Photosynthetic activity*leaf area
treatment
dpt
F
PAM fluorometry 4 dpt
Photosynthetic activity*leaf area at 4 dpt

Fig. S3

Fig. S3: Photosynthetic analysis of Arabidopsis seedlings with *Sv* and/or *Bs* with or without the bacterial SynComs. 6 days post inoculation, the seedlings were washed to remove extraradical hyphae and transferred to 24 well plates. The photosystem II (PS II) quantum yield was measured every 24 h after dark adaptation (F_V/F_M) via PAM fluorometry. Purple/dark blue, lighter colors and black color indicate high, reduced and lack of PS II activity, respectively. A - C) The PS II quantum yield of 5 *At* seedlings/well in absence or presence of *Sv*, *Bs* and/or a bacterial SynCom (*Hv*S or *At*S) at 4 days post transfer (dpt), corresponding to 10 dpi. D) Exemplary time course of PAM fluorometry images from 1 to 7 dpt in the tripartite conditions. E) The photosynthetic activity (F_V/F_M) from 1 – 4 dpt (n = 6). F) The photosynthetic activity*leaf area at 4 dpt (n = 6). *Bs* infection leads to a local reduction of the PS II activity leading to a reduced photosynthetic active leaf area over time. Different letters represent statistically significant differences according to one-way ANOVA and Tukey‘s post-hoc test ($p < 0.05$).

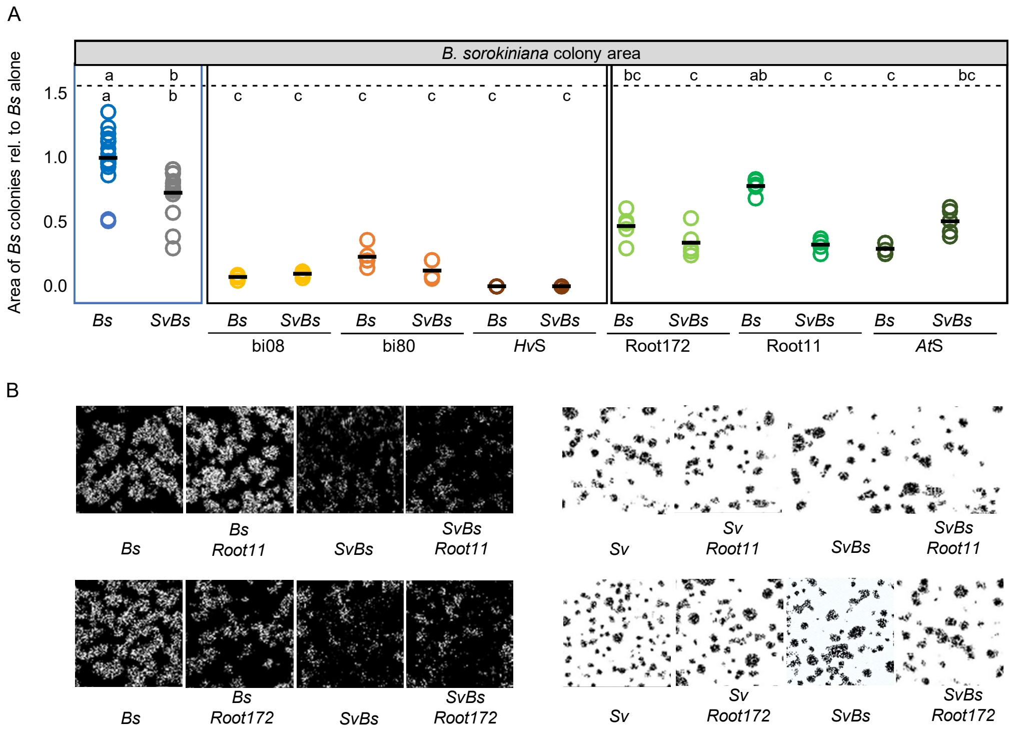

Fig. S4: Microbe-microbe confrontation of *B. sorokiniana*, *S. vermifera* and the bacterial SynComs in absence of the host. A) *Bs* colony area in direct confrontation with *Sv* or bacteria in absence of the host on defined medium relative to *Bs* alone (n = 4 - 16 replicates). B) Pictures of direct confrontation assays. *Bs* colonies (black background) and *Sv* colonies (white background) were filtered using ImageJ (Schneider *et al.*, 2012) and the morphoLipJ plugin (Legland *et al.*, 2016). *Sv* colony area was not negatively affected by the presence of the other microbes. Different letters represent statistically significant differences according to one-way ANOVA and Tukey‘s post-hoc test ($p < 0.05$).

Legland, D., Arganda-Carreras, I., & Andrey, P. (2016). MorphoLibJ: integrated library and plugins for mathematical morphology with ImageJ. Bioinformatics, btw413. doi:10.1093/bioinformatics/btw413

Schneider, C. A., Rasband, W. S., & Eliceiri, K. W. (2012). NIH Image to ImageJ: 25 years of image analysis. *Nature Methods*, *9*(7), 671–675. doi:10.1038/nmeth.2089

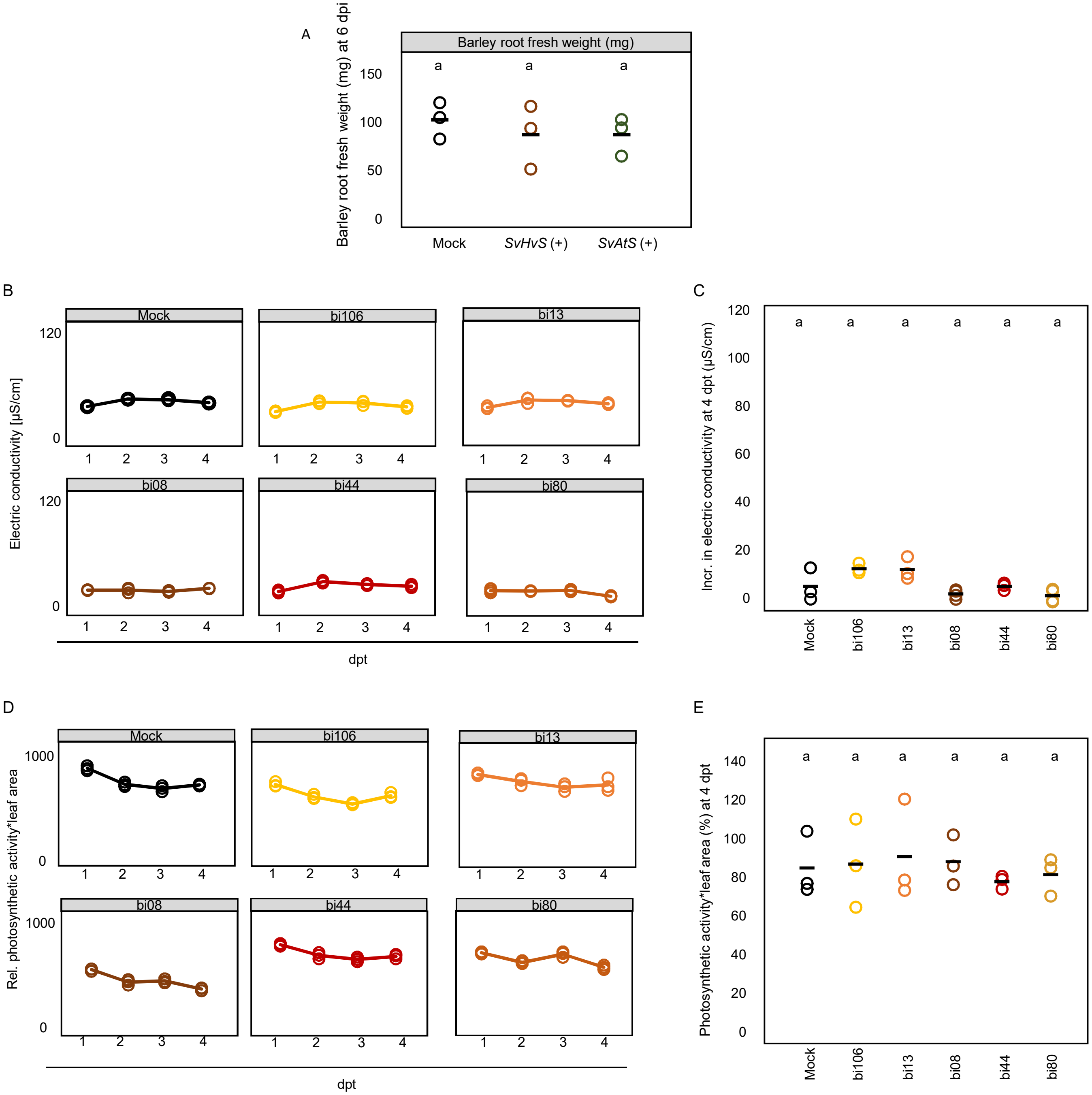

Fig. S5: Plant response to bacteria. A) Root fresh weight of barley seedlings inoculated with *Sv* and the heat-inactivated bacterial SynComs (+). Root weight was measured at 6 dpi (n = 3, mean of 4 plants per replicate shown). No statistically significant difference was detected according to non-parametric Kruskal-Wallis test followed by pairwise Mann–Whitney U-tests for multiple comparisons (FDR adjusted p-value < 0.05). B – E) Arabidopsis seedlings were inoculated with the bacterial strains bi106, bi13, bi08, bi44 or bi80, all derived from the *Hv*SynCom. B) Electric conductivity from 1 to 4 days post transfer (dpt), corresponding to 7 to 10 days post inoculation (n = 3, 5 plants per replicate). C) Total increase in electric conductivity from 1 to 4 dpt (n = 3, 5 plants per replicate). D) Photosynthetic activity (F_V/F_M) from 1 to 4 dpt (n = 3, 5 plants per replicate). E) Photosynthetic activity per leaf area at 4 dpt relative to 1 dpt (n = 3, 5 plants per replicate). No statistically significant difference was detected according to one-way ANOVA and Tukey's post-hoc test ($p < 0.05$) and non-parametric Kruskal-Wallis test followed by pairwise Mann–Whitney U-tests for multiple comparisons (FDR adjusted p-value < 0.05).

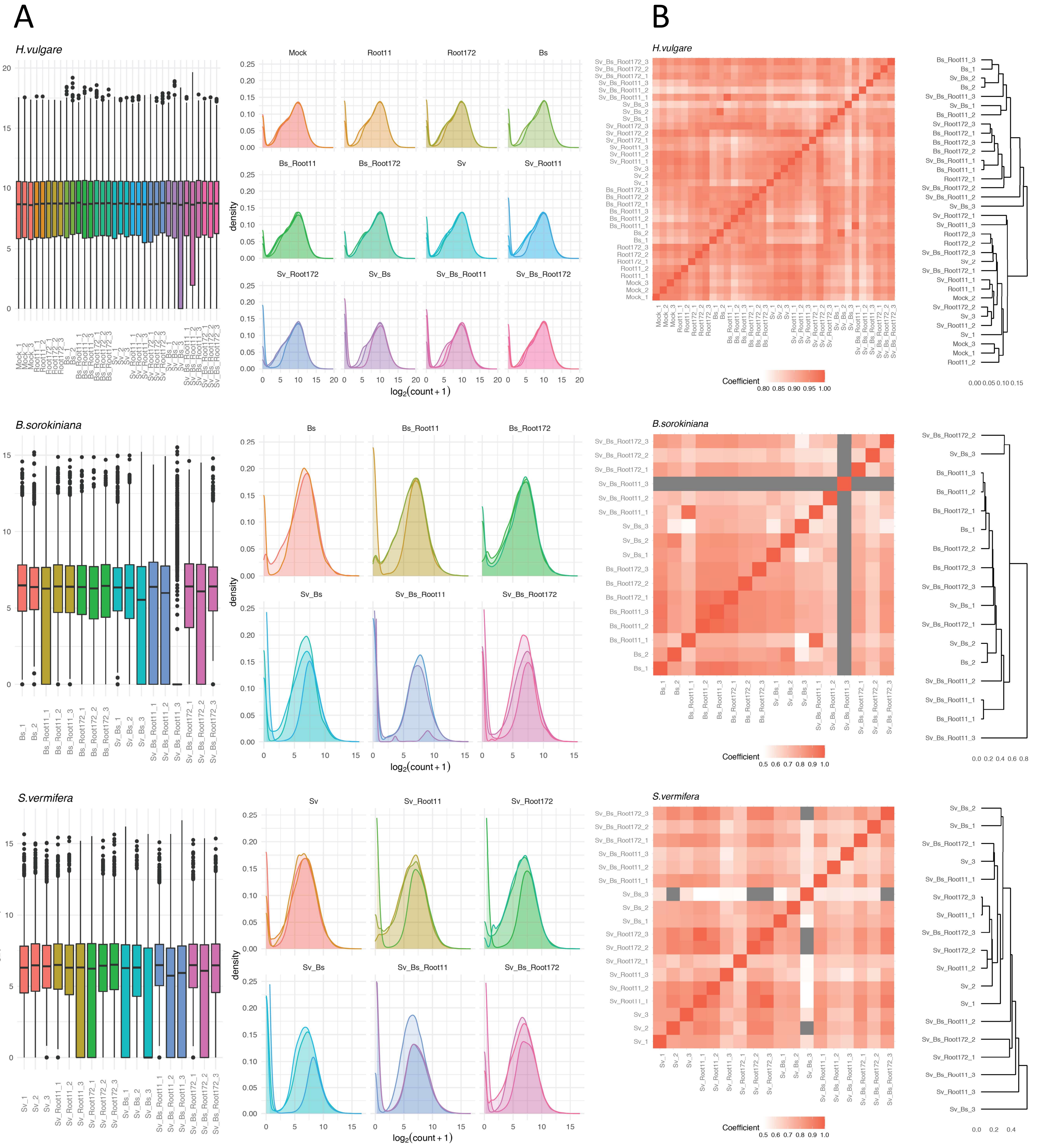

Fig. S6: Assessment of RNA-seq data. A) Distribution and density of the normalised log2 transformed transcript count of genes for three organisms. B) Correlation of transcriptomes of RNA-seq samples. Left part: Adjacent matrix based on the correlation coefficients. Right part: Hierarchical clustering of biological replicates according to the distances of transcriptomic similarities. Grey represents correlation coefficients lower than 0.5.

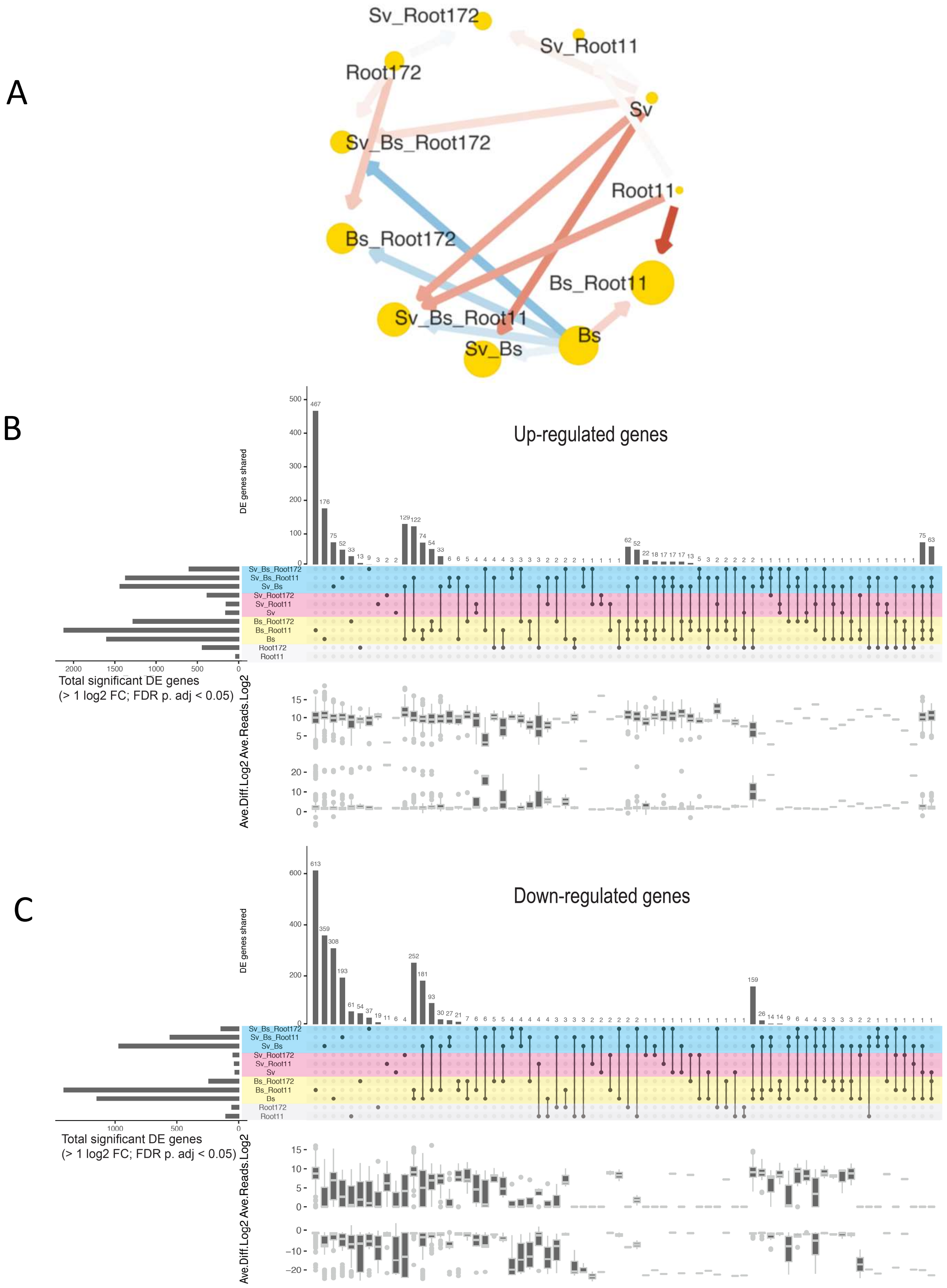

Fig. S7: *H. vulgare* differentially expressed genes. A) Condition-specific differentially expressed genes (DEG, > 1 log2FC; FDR adjusted p-value < 0.05) are compared to barley mock control. Total number of DEG per condition are visualised as a network. The size of the circles corresponds to the total number of DEG. Red and blue arrows connecting the conditions show changes for the number of DEG. See also Fig. 5. B) Up and C) down-regulated genes are separately presented. Horizontal bars: Total number of DEG per condition. Vertical bars: Number of genes unique/shared for top 70 intersections. See Tab. S6.

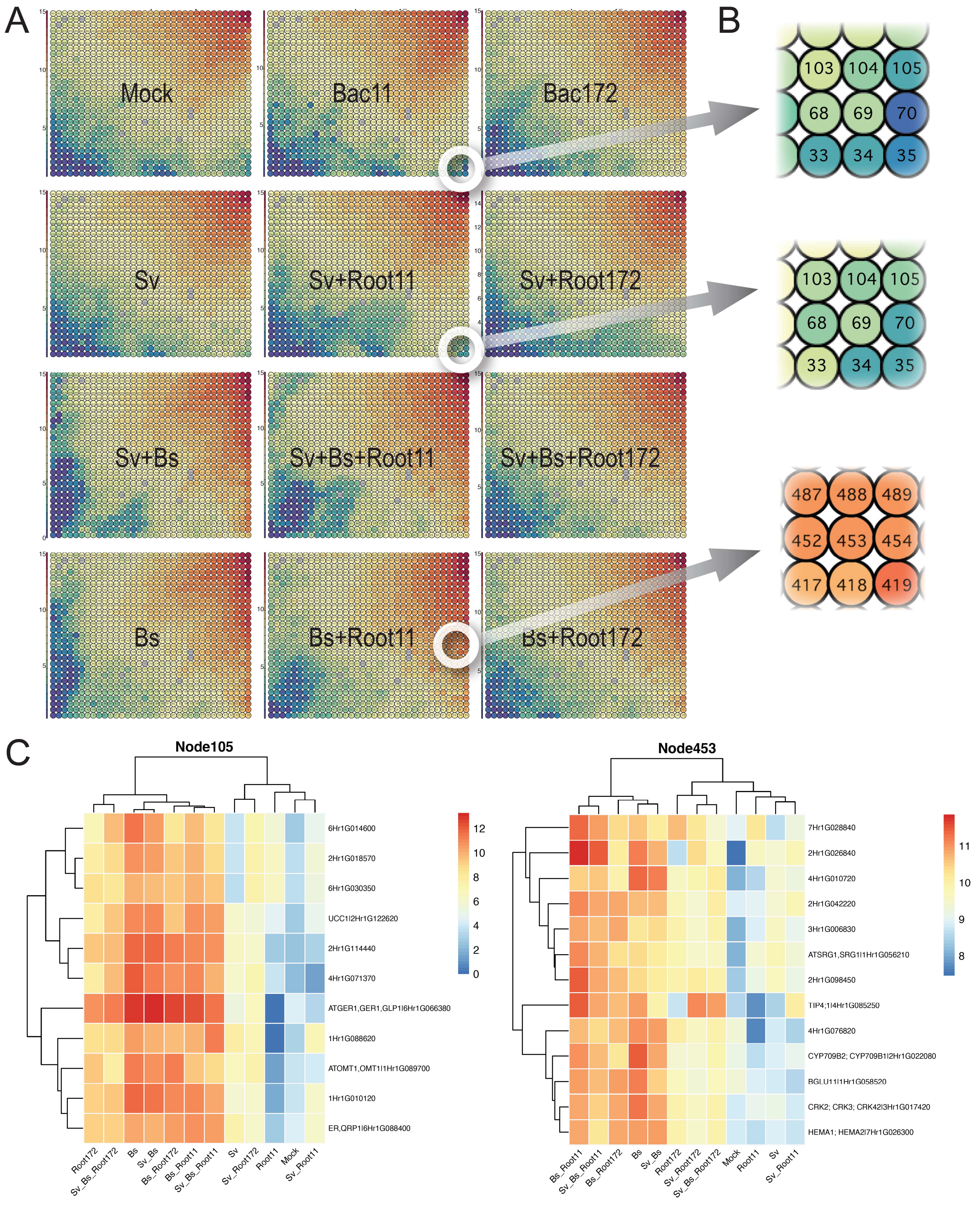
A
Mock
Bac11
Bac172
Sv
Sv+Root11
Sv+Root172
Sv+Bs
Sv+Bs+Root11
Sv+Bs+Root172
Bs
Bs+Root11
Bs+Root172
B
103 104 105
68 69 70
33 34 35
103 104 105
68 69 70
33 34 35
487 488 489
452 453 454
417 418 419
C
Node105
6Hr1G014600
2Hr1G018570
6Hr1G030350
UCC1|2Hr1G122620
2Hr1G114440
4Hr1G071370
ATGER1,GER1,GLP1|6Hr1G066380
1Hr1G088620
ATOMT1,OMT1|1Hr1G089700
1Hr1G010120
ER,QRP1|6Hr1G088400
Root172
Sv_Bs_Root172
Bs
Sv_Bs
Bs_Root172
Bs_Root11
Sv_Bs_Root11
Sv
Sv_Root172
Root11
Mock
Sv_Root11
Node453
7Hr1G028840
2Hr1G026840
4Hr1G010720
2Hr1G042220
3Hr1G006830
ATSRG1,SRG1|1Hr1G056210
2Hr1G098450
TIP4;1|4Hr1G085250
4Hr1G076820
CYP709B2; CYP709B1|2Hr1G022080
BGLU11|1Hr1G058520
CRK2; CRK3; CRK42|3Hr1G017420
HEMA1; HEMA2|7Hr1G026300
Bs_Root11
Sv_Bs_Root11
Bs_Root172
Bs
Sv_Bs
Root172
Sv_Root172
Sv_Bs_Root172
Mock
Root11
Sv
Sv_Root11

Fig. S8

Fig. S8: Genome-wide transcriptomic dynamics of *H. vulgare* per condition. A) Trained Self-Organizing Maps (SOM, Tatami maps) showing barley global transcriptomic trends. Colors indicate the averaged log2 read count of replicates from each condition. Each circle represents a node (IDs 1 to 1015). Single nodes contain approximately 10 to 100 genes. The SOM resulted in similarly-expressed genes separated into high, medium, and low expressed groups. The highly transcribed genes are clustered at the top right corner (red) and the lowly transcribed groups at the bottom left corner (blue). Barley inoculated with *S. vermifera* (*Sv*) exhibited similar patterns to barley mock. The presence of the pathogen (*Bs*) was a major factor driving responses in the host, which was consistent with the dynamics of DEG shown in Fig. 5. There were additional effects of the co-inoculated bacteria on barley (Root11 and Root172). The shape of the lowly transcribed clusters shifted in co-inoculated roots with the bacterial strains (e.g. *Bs* vs *Bs*+Root11 or *Bs*+Root172). B) Double-circles (i.e. white doughnuts) on Tatami maps indicate the location of highly regulated gene groups and such gene groups are magnified. C) Examples of highly regulated genes (FDR adjusted $p < 0.05$) present in particular nodes. The high and low log2 gene expression is displayed in red and blue respectively. Gene identification number with corresponding annotations (if there is any) are presented on Y-axis. Node 105 contains similarly lowly expressed genes for barley mock, bacterium 11, and *S. vermifera* (Mock, Root11, Sv_Root11). Node 453 shows highly expressed genes for *B. sorokiniana* with bacterium 11 (Bs_Root11). See Tab. S3 for details.

GO enrichment analysis (up-regulated barley genes)

Cross comparison of SEA (SEACOMPARE)

AgriGO V2 doi: 10.1093/nar/gkx382

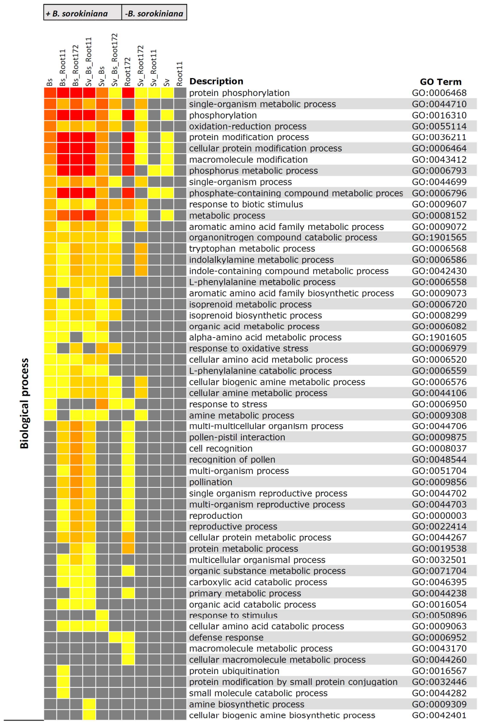

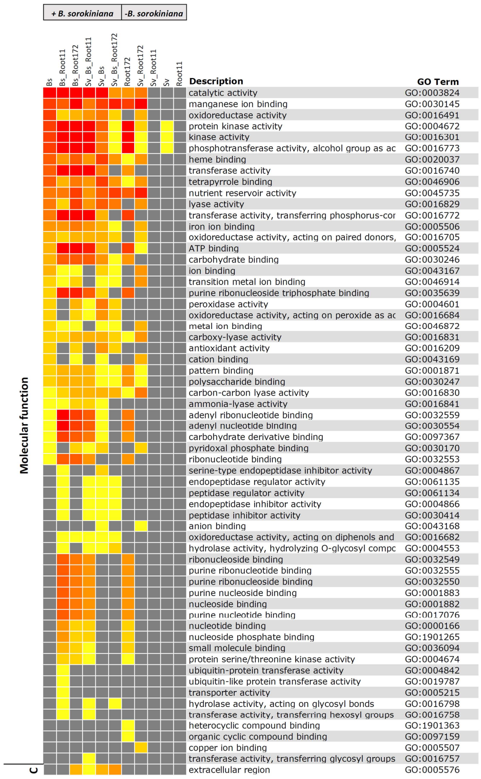

Color code: red - most significant, yellow - significant, grey -not significant

Differentially expressed barley genes compared to mock.

C: Cellular Component

GO enrichment analysis (down-regulated genes)

Cross comparison of SEA (SEACOMPARE)

AgriGO V2 doi: 10.1093/nar/gkx382

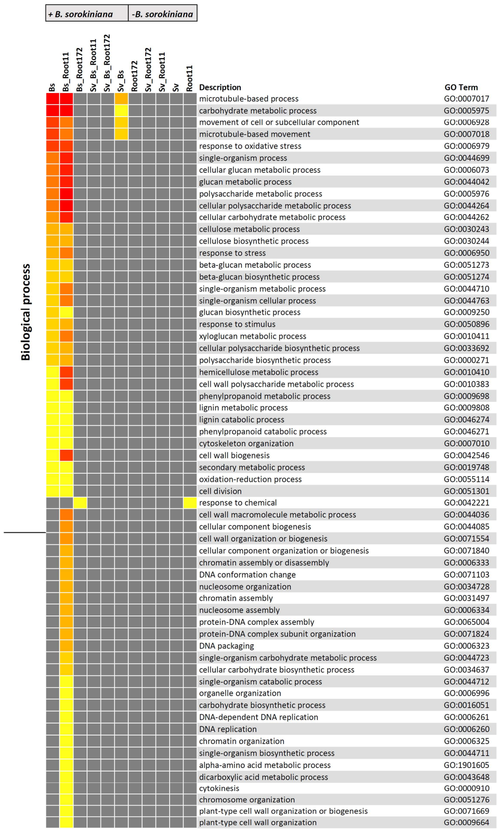

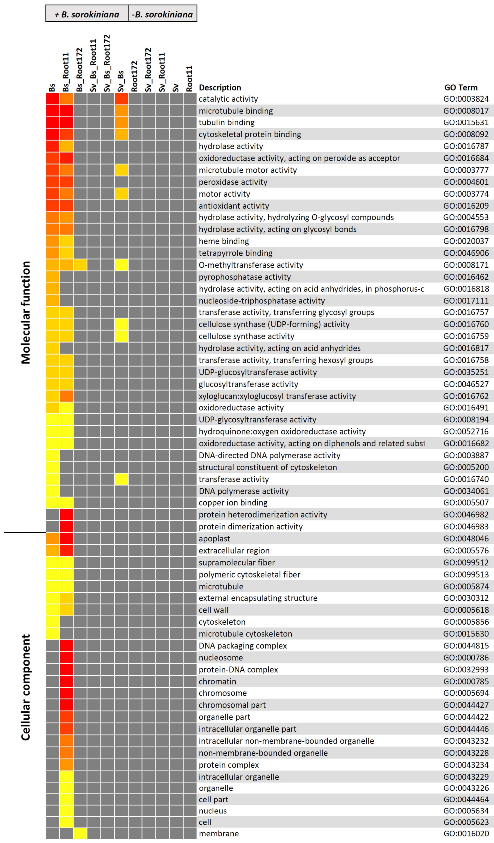

Color code: red - most significant, yellow - significant, grey -not significant

Differentially expressed barley genes compared to mock.

Fig. S9: GO enrichment analysis (up- and down-regulated barley genes)

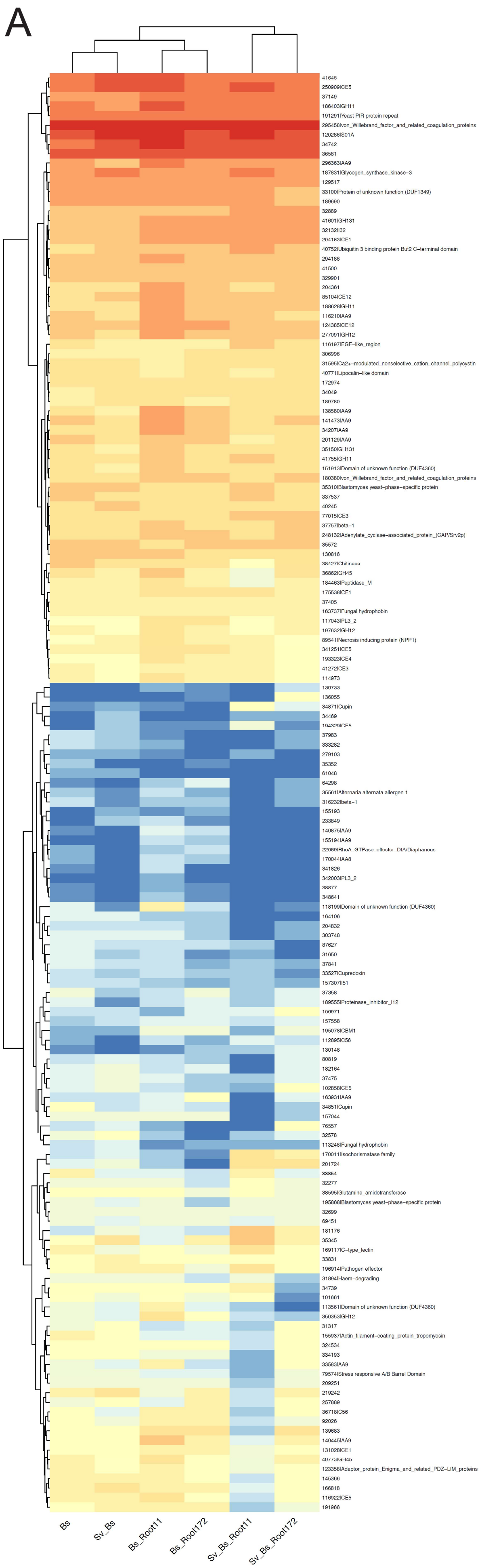

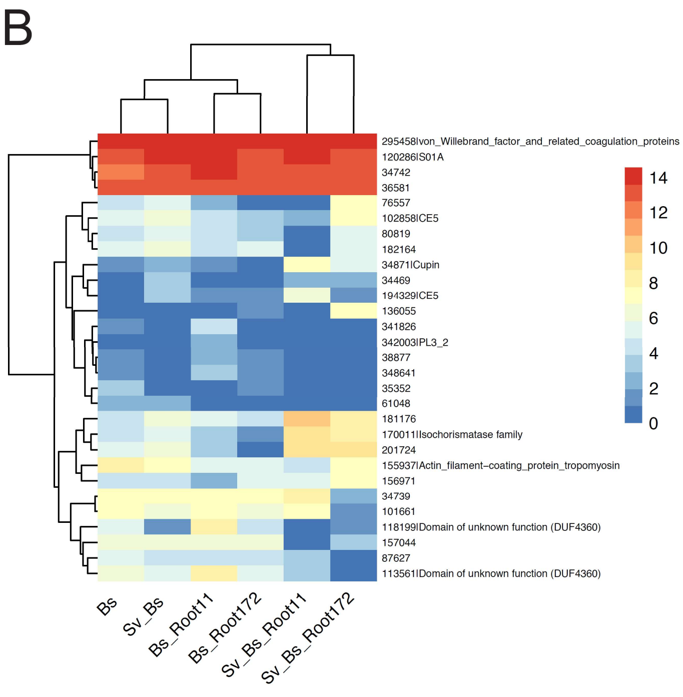

Fig. S10: Expression of genes coding for effectors in *B. sorokiniana*. A) Averaged log2 read count of genes under the conditions. Y-axis shows JGI Protein IDs with corresponding annotations. B) Averaged log2 read count of genes with high loadings (see Methods). Y-axis shows JGI Protein IDs with corresponding annotations if there is any. See Tab. S9.

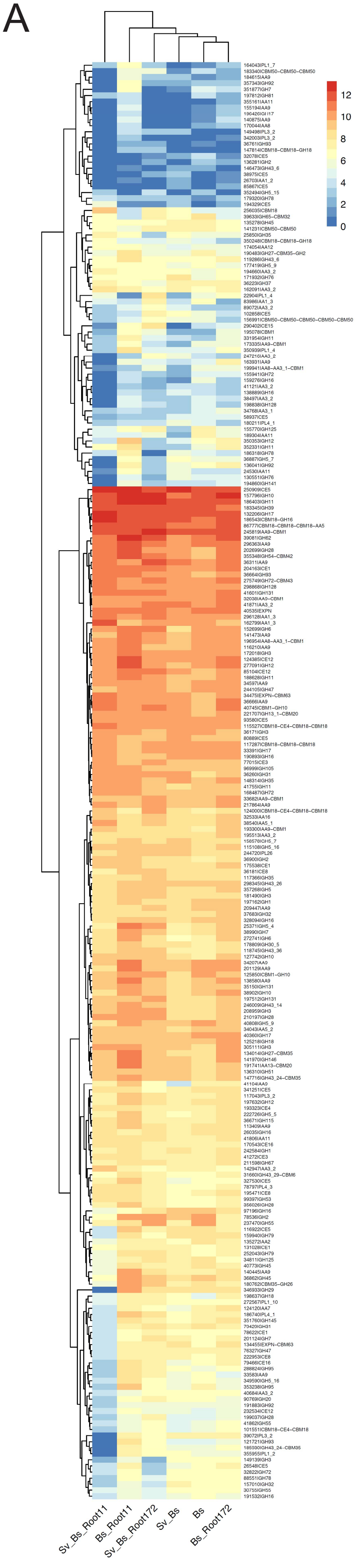

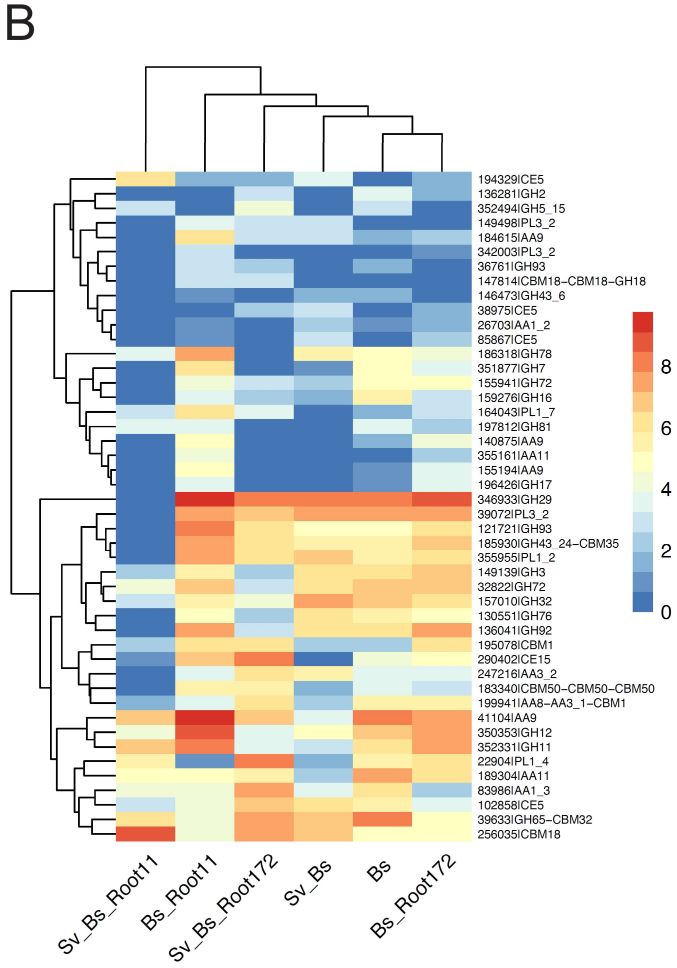

Fig. S11: Expression of genes coding for CAZymes predicted to be secreted in *B. sorokiniana*. A) Averaged log2 read count of genes under the conditions. Y-axis shows JGI Protein IDs with corresponding annotations. B) Averaged log2 read count of genes with high loadings (see Methods). Y-axis shows JGI Protein IDs with corresponding annotations if there is any. See Tab. S9.

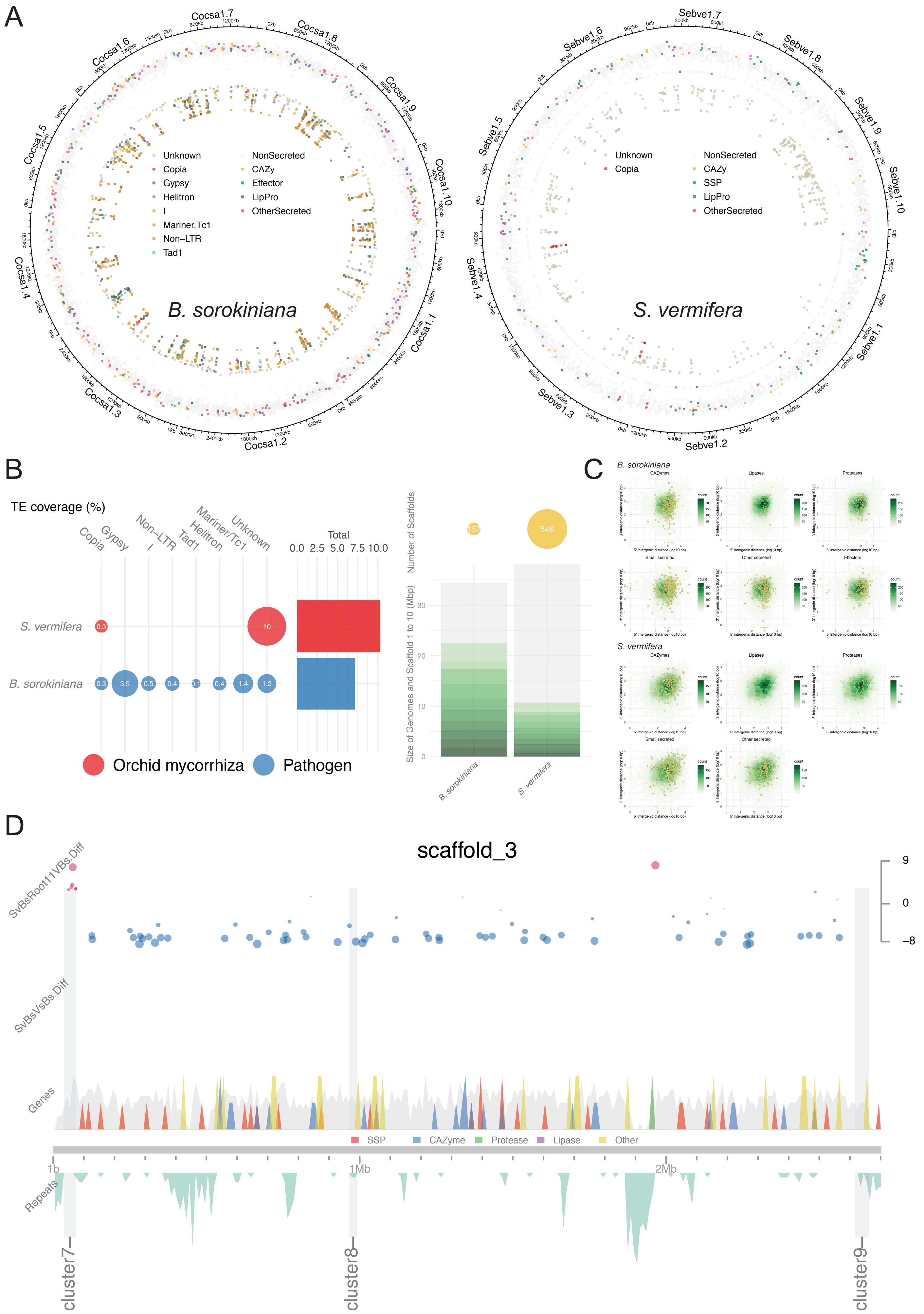
A
B. sorokiniana
S. vermifera
Unknown
Copia
Gypsy
Helitron
I
Mariner.Tc1
Non−LTR
Tad1
NonSecreted
CAZy
Effector
LipPro
OtherSecreted
SSP
B
TE coverage (%)
Copia
Gypsy
I
Non−LTR
Tad1
Helitron
Mariner/Tc1
Unknown
Total
S. vermifera
B. sorokiniana
Orchid mycorrhiza
Pathogen
Number of Scaffolds
Size of Genomes and Scaffold 1 to 10 (Mbp)
C
D
scaffold_3
SvBsRoot11VBs.Diff
SvBsVsBs.Diff
Genes
SSP
CAZyme
Protease
Lipase
Other
Repeats
cluster7
cluster8
cluster9

Fig. S12

Fig. S12: Genomic features of *B. sorokiniana* and *S. vermifera*. A) The genomic location of genes and transposable elements (TEs) are visualised with the largest 1 to 10 scaffolds from the genome assemblies. Hanabi plots (fireworks in Japanese) contains three rings. Outer ring: The size of scaffold 1 to 10 presented clock-wise starting from 3 o'clock. Colors of Scaffold 1 to 10 are from dark grey to light grey. The boxes next to “fungal names + scaffold ID” represents the length of the scaffolds. Approximate locations of genomic features can be seen with the small rulers aligned in the outer ring. Middle ring: The genomic locations of all genes based on JGI GFF files. Genes coding for theoretically secreted proteins (CAZymes, SSPs, lipases, proteases) are in color. Other genes coding for non-secreted (i.e. intracellular) proteins are in grey. Inner ring: The genomic locations of TE families and unidentified repeats. Repeat sequences (>50 bases with >10 occurrences in a genome) were identified. Vertical axis for the density of genes/TEs in the rings: The mean distance of neighboring genes or TEs in log2. If distances between genes/TEs are short, dots (i.e. the locations of genes and TEs) go towards the centre of plots. If distances between genes/TEs are long, dots go towards the outer circle (it gives a sense of how densely localized or dispersed genes/TEs are). See Tab. S10 for details. B) TE content and scaffolds in the genome assemblies. Left panel: Coverage of transposable elements in the genomes. The size of the bubbles corresponds to the percentage of TE coverage in the genomes. Right panel: Genome size and the number of scaffolds. The bars in grey indicate the genome size. Individual green sections shows the largest scaffolds 1 to 10. The circle size corresponds to the number of total scaffolds. The ecological lifestyle is in color. C) Intergenic distances of genes for secreted proteins (i.e. intergenic distance = gene to gene distance). Proteins predicted to be secreted are categorised into CAZymes, proteases, lipases, the rest of secreted protein, effectors, and a subcategory for small secreted proteins (< 300 amino acids). Yellow points: Intergenic 5’ and 3’ distances of individual genes. Green tiles: Density of intergenic distances of all genes present in a genome. Genes tend to be gathered at the centre of the maps, showing average intergenic distances. Genes nearby a cluster of transposable elements tend to show long intergenic distances (see top right corner) where new functions of genes might be evolved due to the transposition. See Table S11. D) Visual integration of multi-omics showing highly regulated biosynthetic gene clusters in *B. sorokiniana*. Omics data (transcriptome, secretome, repeatome and genome) are combined and visualised. Scaffold 3 from the genome assembly is presented for example. Grey vertical bars: Biosynthetic gene clusters. Top panel: Significantly regulated genes under conditions. The size of circles and colors correspond to differential transcription levels in log2. Middle panel: The genomic locations and density of all genes (grey) and gene for secreted proteins (colors). The scaffold size of a genome assembly is shown as a grey horizontal bar. Bottom panel: The genomic location and density of total and individual TE families. See Tab. S12 for details.

Supplementary Methods to Fig. S12

Multi-omics integration and visualization for fungi. Secreted proteins were predicted using the method described previously (Pellegrin *et al*., 2015). CAZy annotations were provided from CAZy team (www.cazy.org). Transposable element (TE) identification was performed with Transposon Identification Nominative Genome Overview (TINGO; Morin *et al*., 2019). We predicted biosynthetic gene clusters with antiSMASH 5.1 (Medema *et al*., 2011). Differential expression of genes was calculated with the control, *B. sorokiniana* alone grown in barley using DESeq2 (Love *et al*., 2014). We excluded genes showing either very low raw reads or adjusted p value (FDR) larger than 0.05. Differentially expressed genes coding for effectors were obtained from the previous study (Sarkar *et al*., 2019). Output files obtained from the various analyses above and functional annotations from JGI MycoCosm were cleaned, sorted, combined and visualized using a set of custom R scripts, Visually Integrated Numerous Genres of Omics (VINGO; Looney *et al*., 2021) incorporating R package karyoploteR (Gel & Serra 2017). Also, we located genomic features (i.e. genes, predicted secretome, transposable elements) in the largest scaffold 1 to 10 in a circular manner (Hanabi plots) with Syntenic Governance Overview (SynGO; Hage *et al*., 2021) incorporating R package Circlize for visualization (Gu *et al*., 2014).

Visual intergenic distances in genomes with statistics. Intergenic distances in the genomes were calculated based on the study (Saunders *et al*. 2014). The original scripts are obtained from https://github.com/Adamtaranto/density-Mapr. Theoretically secreted proteins were determined with Secretome pipeline mentioned above. The results were visualized using a visual pipeline SynGO (Hage *et al*., 2021). The mean TE-gene distances were calculated from; (i) the locations of observed genes and TEs; and (ii) random "null hypothesis" genome models made by randomly reshuffling the locations of genes. The distribution of genomic features was purely random for null models and there was no association between the locations of genes and repeat elements. The probability (p-value) of mean TE-gene distances was calculated based on a normal distribution of 10,000 null hypothesis models. The process was performed with R package, regioneR (Gel *et al*., 2016).

Gel & Serra. (2017). karyoploteR: an R/Bioconductor package to plot customizable genomes displaying arbitrary data. Bioinformatics, 31–33. doi:10.1093/bioinformatics/btx346

Gel, B. et al. regioneR: an R/Bioconductor package for the association analysis of genomic regions based on permutation tests. Bioinformatics 32, 289–91 (2016).

Gu et al. circlize implements and enhances circular visualization in R. Bioinformatics 30, 2811–2812 (2014).

Hage, H. *et al.* Gene family expansions and transcriptome signatures uncover fungal adaptations to wood decay. *Environ. Microbiol.* (2021).

Looney, B. *et al.* Comparative genomics of Russulales Evolutionary priming and transition to the ectomycorrhizal habit in an iconic lineage of mushroom-forming fungi : is preadaptation a requirement? Comparative genomics of Russulales. bioRxiv (2021).

Love, M. I., Huber, W., and Anders, S. (2014). Moderated estimation of fold change and dispersion for RNA-seq data with DESeq2. Genome Biol. 15, 550. doi:10.1186/PREACCEPT-8897612761307401.

Medema, M. H. et al. antiSMASH: rapid identification, annotation and analysis of secondary metabolite biosynthesis gene clusters in bacterial and fungal genome sequences. Nucleic Acids Res. 39, W339-46 (2011).

Morin, E. et al. Comparative genomics of Rhizophagus irregularis, R. cerebriforme, R. diaphanus and Gigaspora rosea highlights specific genetic features in Glomeromycotina. New Phytol. 222, 1584–1598 (2019).

Pellegrin, C., Morin, E., Martin, F. M. & Veneault-Fourrey, C. Comparative Analysis of Secretomes from Ectomycorrhizal Fungi with an Emphasis on Small-Secreted Proteins. Front. Microbiol. 6, 1278 (2015).

Sarkar, D. et al. The inconspicuous gatekeeper: endophytic Serendipita vermifera acts as extended plant protection barrier in the rhizosphere. New Phytol. 224, 886–901 (2019).

Saunders, Diane GO, et al. "Two-dimensional data binning for the analysis of genome architecture in filamentous plant pathogens and other eukaryotes." Plant-Pathogen Interactions: Methods and Protocols (2014): 29-51.

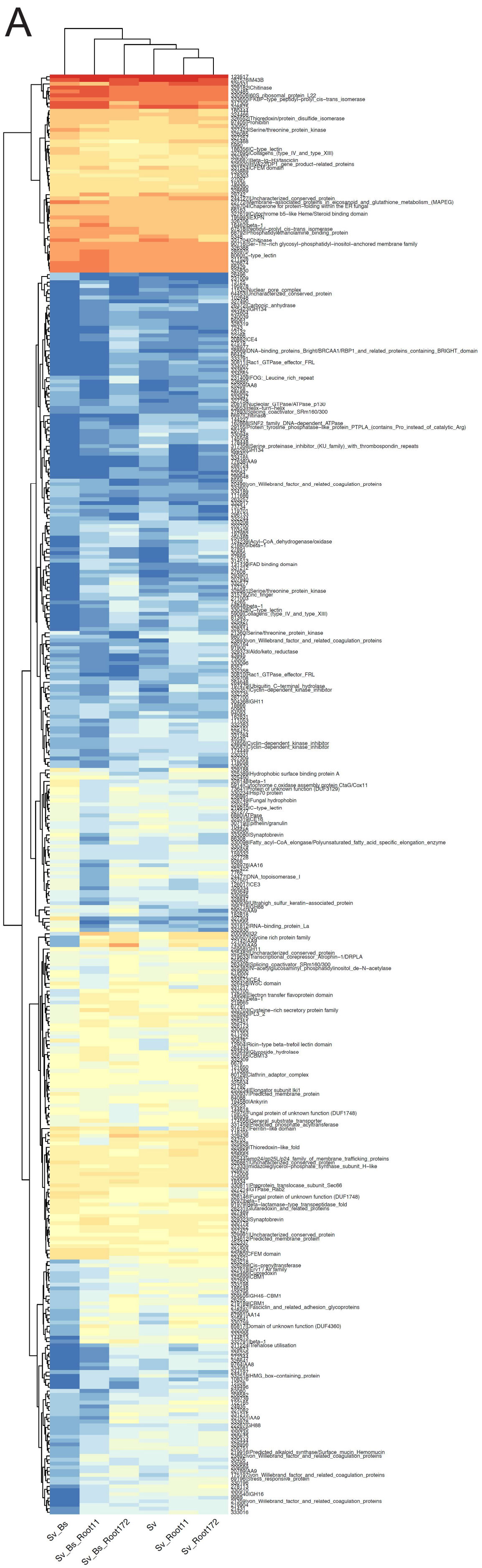

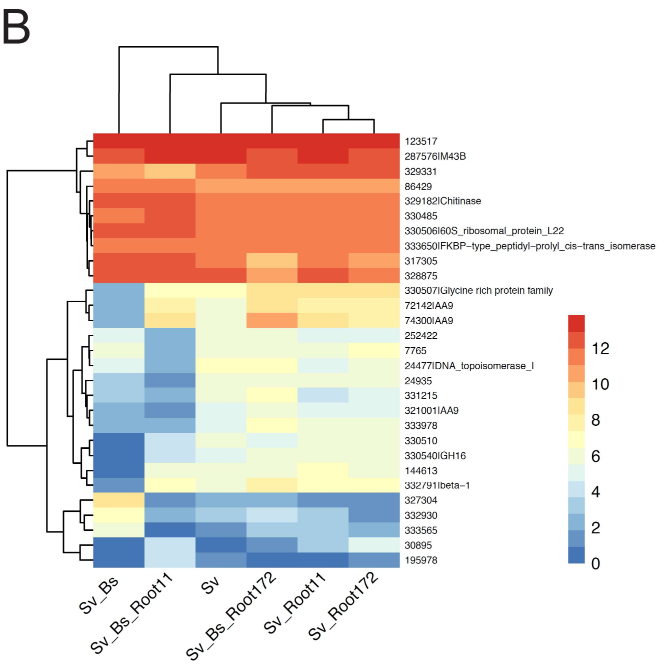

Fig. S13. Expression of genes coding for effectors in *S. vermifera*. A) Averaged log2 read count of genes under the conditions. Y-axis shows JGI Protein IDs with corresponding annotations. B) Averaged log2 read count of genes with high loadings (see Methods). Y-axis shows JGI Protein IDs with corresponding annotations if there is any. See Tab. S9.

A

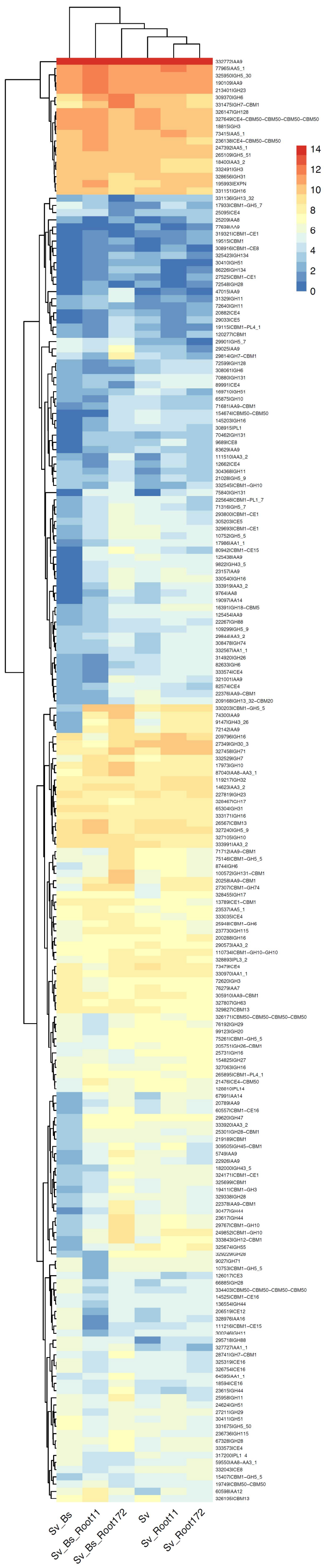

B

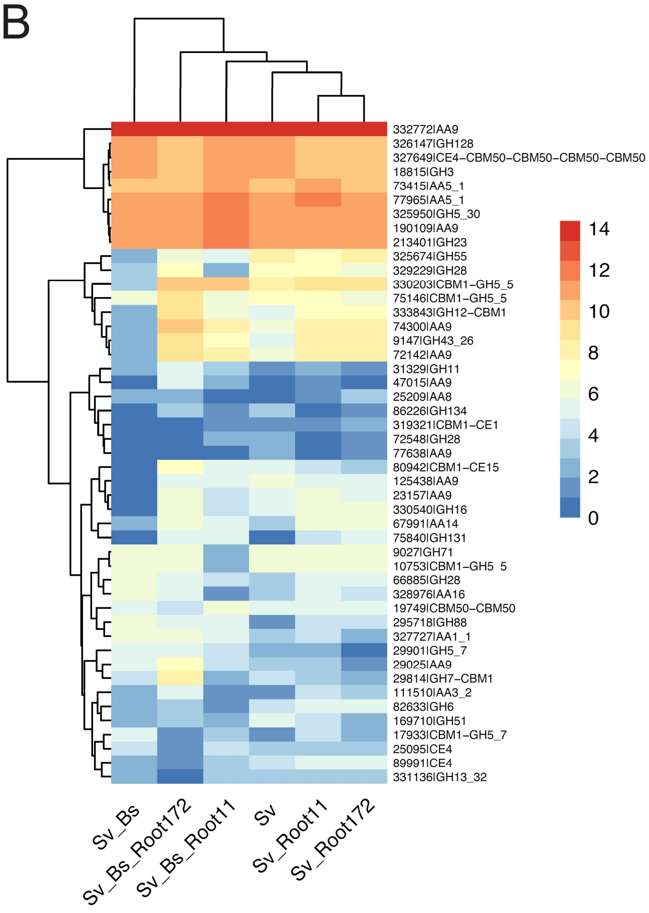

Fig. S14: Expression of genes coding for CAZymes predicted to be secreted in *S. vermifera*. A) Averaged log2 read count of genes under the conditions. Y-axis shows JGI Protein IDs with corresponding annotations. B) Averaged log2 read count of genes with high loadings (see Methods). Y-axis shows JGI Protein IDs with corresponding annotations if there is any. See Tab. S9.
