# Supplementary material for: The fungal root endophyte Serendipita vermifera displays inter-kingdom synergistic beneficial effects with the microbiota in Arabidopsis thaliana and barley
Source: ISME J. 2021 Oct 22;16(3):876–89. doi: 10.1038/s41396-021-01138-y (PMC8857181; doi:10.1038/s41396-021-01138-y)
Supplement: Supplementary file 2 — Table S1 [file 41396_2021_1138_MOESM2_ESM.pdf]

Table S1: List of primers used for qRT-PCR in this study

Name	Sequence (5‘ → 3‘)	Gene ID / Reference
SvTEF-Fw<br>*SvTEF*-Rev	ATCCCAAGCAAGCCAATGTG<br>TGCCGTCAGTCTTCTCAACA	Transcript Id 325682<br>JGI MycoCosm
BsTEF-Fw<br>*BsTEF*-Rev	CGCCGTACCGGAAAGTCTG<br>GGCGAAACGACCAAGAGGA	Transcript Id 29877<br>JGI MycoCosm
*HvUbi*_Fw<br>*HvUbi*_Rev	ACCCTCGCCGACTACAACAT<br>CAGTAGTGGCGGTCGAAGTG	(Sakar et al., 2019)
*HvPR10*_Fw<br>*HvPR10*_Rev	GGAGGGCGACAAGGTAAGTG<br>CGTCCAGCCTCTCGTACTCT	(Sakar et al., 2019)
*AtUbi*_Fw<br>*AtUbi*_Rew	CCAAGCCGAAGAAGATCAAG<br>ACTCCTTCCTCAAACGCTGA	AT3G62250
*At1g58420*_Fw<br>*At1g58420*_Rev	TGGGAGCGGTTACGGTTAAAGG<br>AGCCATTGTCATCCCAACACTCG	At1g58420
